# Activation of the Sympathetic Nervous System Promotes Blood Pressure Salt-Sensitivity in C57BL6/J Mice

**DOI:** 10.1161/HYPERTENSIONAHA.120.16186

**Published:** 2020-11-16

**Authors:** Ailsa F. Ralph, Celine Grenier, Hannah M. Costello, Kevin Stewart, Jessica R. Ivy, Neeraj Dhaun, Matthew A. Bailey

**Affiliations:** From the University/BHF Centre for Cardiovascular Science, The Queen’s Medical Research Institute, The University of Edinburgh, United Kingdom.

**Keywords:** blood pressure, hypertension, mice, natriuresis, sympathetic nervous system

## Abstract

Global salt intake averages >8 g/person per day, over twice the limit advocated by the American Heart Association. Dietary salt excess leads to hypertension, and this partly mediates its poor health outcomes. In ≈30% of people, the hypertensive response to salt is exaggerated. This salt-sensitivity increases cardiovascular risk. Mechanistic cardiovascular research relies heavily on rodent models and the C57BL6/J mouse is the most widely used reference strain. We examined the effects of high salt intake on blood pressure, renal, and vascular function in the most commonly used and commercially available C57BL6/J mouse strain. Changing from control (0.3% Na^+^) to high salt (3% Na^+^) diet increased systolic blood pressure in male mice by ≈10 mm Hg within 4 days of dietary switch. This hypertensive response was maintained over the 3-week study period. Returning to control diet gradually reduced blood pressure back to baseline. High-salt diet caused a rapid and sustained downregulation in mRNA encoding renal *NHE3* (sodium-hydrogen-exchanger 3) and *EnaC* (epithelial sodium channel), although we did not observe a suppression in aldosterone until ≈7 days. During the development of salt-sensitivity, the acute pressure natriuresis relationship was augmented and neutral sodium balance was maintained throughout. High-salt diet increased ex vivo sensitivity of the renal artery to phenylephrine and increased urinary excretion of adrenaline, but not noradrenaline. The acute blood pressure–depressor effect of hexamethonium, a ganglionic blocker, was enhanced by high salt. Salt-sensitivity in commercially sourced C57BL6/J mice is attributable to sympathetic overactivity, increased adrenaline, and enhanced vascular sensitivity to alpha-adrenoreceptor activation and not sodium retention or attenuation of the acute pressure natriuresis response.

## Abstract 2

Supplemental Digital Content is available in the text.

Globally, individual salt (sodium chloride) intake is estimated at >8 g/day,^1–3^ more than twice the upper limit recommended by the American Heart Association.^4^ This habitual salt excess is associated with a range of poor health outcomes, including autoimmunity,^5^ cardiovascular and chronic kidney disease,^6^ dementia,^7^ and gastrointestinal cancers.^8^ Hypertension makes an important contribution to many of these and the association between salt intake and blood pressure (BP) is well documented.^9^ There is an exaggerated rise in BP in response to salt in ≈30% of people, who are categorized as salt-sensitive.^10^ Even if BP is within the normal range, salt-sensitivity increases mortality risk^11^ and is an independent cardiovascular risk factor.^12^

The physiological and molecular mechanisms of salt-sensitivity are not fully defined. A kidney-centered hypothesis suggests that salt-sensitivity is a failure of the acute pressure natriuresis mechanism, which normally acts to maintain extracellular fluid volume homeostasis and buffer BP against dietary salt excess.^13^ A vascular-centered hypothesis suggests that salt-sensitivity is a result of a failure of peripheral vasodilation to accommodate increased salt intake and extracellular fluid volume expansion.^14^ A brain-centered hypothesis argues that abnormal sympathetic outflow, particularly to the kidney and vasculature, is the primary defect underlying salt-sensitive hypertension.^15^ Overall the physiological basis of salt-sensitivity remains contentious.^16,17^

Our understanding of salt-sensitivity has been deepened by experiments in animals.^18,19^ The capacity to manipulate the rodent genome has identified new molecular pathways through which high-salt diet can increase BP. These include primary alterations within the immune, neurological, renal, or vascular systems.^20–23^ Such studies rely on a salt-resistant reference strain, a control animal in which BP is not increased by high-salt diet. C57BL6 mouse strains are widely used in this context, both as an inbred experimental mouse strain and as the congenic background for transgenic lines. Like humans, C57BL6 mice have a single renin gene, resulting in a lower resting BP than other inbred strains with 2 renin genes.^24^ C57BL6 mice (chiefly J and N strains) are often considered salt-resistant, as several studies, including our own, indicate that high-salt diet alone does not increase systolic BP in either the parent strain or transgenic wild-type mice congenic on this background.^22,25,26^ However, others report salt-sensitivity.^27,28^

Given the importance of C57BL6/J mice in hypertension research, the current study used radio-telemetry to define the BP response to high-salt diet in C57BL6/J mice commercially sourced from Charles River, United Kingdom. Demonstrating salt-sensitivity, we performed detailed renal, vascular, and hormonal measurements to identify the physiological processes that might contribute to the sustained rise in BP in these animals.

## Methods

The data that support the findings of this study are available from the corresponding author upon reasonable request.

Please see the Data Supplement for experimental details.

Adult male C57BL6/J mice (Charles River, United Kingdom), aged 10 to 12 weeks, were used in these experiments, which were performed between 2016-2019. Experiments were performed in accordance with the UK’s Animals (Scientific Procedures) Act under a UK Home Office Project Licence. All protocols were reviewed by the University’s Animal Welfare and Ethics Review Board before experimentation. Mice were housed in stock-holding rooms with an ambient temperature of 21±1 °C or in procedural rooms with an ambient temperature of 24±1 °C; humidity was controlled at 50±10% and all rooms operated on a 12-hour light-dark cycle (light period 0700-1900 local time). Mice were housed in groups excepting the radio-telemetry and metabolic cage studies in which mice were housed singly, with access to bedding and enrichment. Unless otherwise specified, mice were killed by cervical dislocation. Mice had free access to water and food throughout. The control diet had 0.3% Na and 0.7% K by weight (RM1 diet, Special Diet Services, United Kingdom); the high-salt diet contained 3% Na and 0.6% K by weight (829504; Special Diet Services, United Kingdom). For cross-sectional experiments, mice were randomized into diet treatment group and the experiments were performed with a single-blind to group allocation.

### Data and Statistics

Data are expressed as mean±SD or as median and range. The sample number (n) for individual experiments is indicated in the figure legends. Cosinor analysis of radio-telemetry data^29^ is detailed in the Data Supplement. Statistical analysis was performed using GraphPad Prism v8.4. Before statistical analysis, normality-testing was performed using the Shapiro-Wilk test. Normally distributed datasets were then analyzed by *t* test, 1-way ANOVA, or 2-way ANOVA, with or without repeated measures, as appropriate. For ANOVA, Holm-Sidak post-tests were used for planned comparisons only when the main effect *P* value was <0.05. Non-normally distributed data were compared using Kruskal-Wallis test for multiple comparisons or Wilcoxon Signed Rank test for matched pairs. Statistical analysis details are provided in the figure legends; absolute *P* values for planned comparisons are reported to 3decimal places.

## Results

### BP, Heart Rate, and Activity

**Figure 1.**
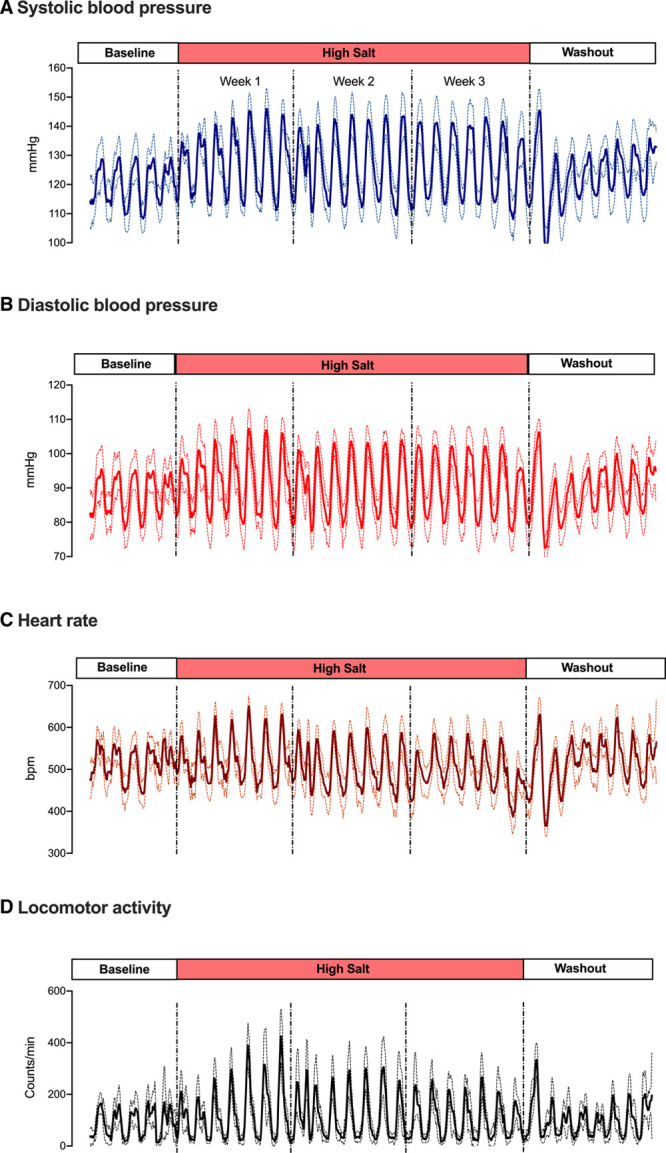
**Cardiovascular parameters and activity in male C57BL6/J mice.** **A**, Systolic blood pressure, (**B**) diastolic blood pressure, (**C**) heart rate and (**D**) locomotor activity recorded by radio-telemetry devices implanted into the carotid artery of male C57BL6/J mice (n=7).Data were acquired every 20 min over a longitudinal protocol (Figure S1) incorporating control diet (0.3% Na) baseline; 21 d of high salt (3% Na) and a washout phase when mice were returned to 0.3% Na diet. Data are presented as 5-hour rolling averages (group mean solid line) ±SD (dashed lines).

Systolic and diastolic BP were measured in conscious, unrestrained mice in a longitudinal experiment encompassing a control diet baseline, 21 days of high-salt diet, and a washout period when animals returned to control diet. The transition to high salt increased both systolic and diastolic BP (ANOVA main effect of diet *P*<0.001), reaching steady-state after 4 days (Figure 1). As salt-sensitivity is often accompanied by the development of nondipping BP,^30^ we, therefore, used cosinor analysis to show that the increase in systolic BP in response to high-salt diet was associated with diurnal abnormalities. However, BP amplitude, reporting the difference between the peak and nadir, was increased rather than attenuated (Figure S2 in the Data Supplement), and this enhanced BP variation was driven by an exaggerated response to salt during the active-phase rather than an enhanced sleep-phase BP dip (Figure S3).

Heart rate increased transiently during the first week of high salt feeding as did the amplitude of daily variation (Figure 1 and Figure S4) due to asymmetrical responses during active and inactive phases of the day (Figure S3). Although overall daily activity was unaffected by diet, rhythmic amplitude increased (Figure 1 and Figure S5) because night-time, active-phase activity was significantly higher during the high salt phase (Figure S3).

### Renal Sodium and Water Handling

**Figure 2.**
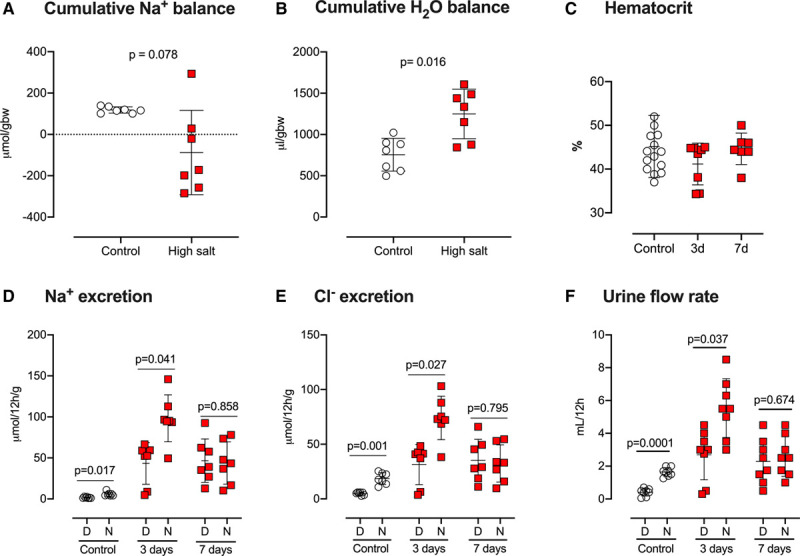
**Male C57BL6/J mice (n=7; 10–12 wk old) were individually housed in metabolism cages for measurement of food/water intake and urine/fecal output over 7 d of the control diet (0.3% Na; open circles), followed by 7 d of high-salt diet (3% Na; red squares).** **A**, Cumulative sodium balance and (**B**) cumulative water balance were calculated for each 7-d period, presented as individual data points with median±interquartile range, and analyzed nonparametrically by Kruskal-Wallis test. **C**, Hematocrit, measured from arterial blood in male C57BL6/J mice fed either control diet (open circles; n=14) or high-salt diet (red squares) for 3 d (n=8) and 7 d (n=7). Individual data points, with mean±SD, were analyzed by 1-way ANOVA and groups were not significantly different. **D**, sodium excretion; (**E**) chloride excretion and (**F**) urine flow rate during the day (D: inactive phase 7 am–7 pm) and night (N: active phase 7 pm–7 am). Individual data points and group mean±SD are shown and analyzed by repeated-measures 2-way ANOVA for main effects of time of day and diet. *P* values for planned comparisons are given, made Holm-Sidak testing.

In balance studies, daily food intake was unaffected by the transition from control to high-salt diet. Consequently, sodium intake increased 10-fold, matched by an increase in urinary sodium excretion (Figures S6). There was no evidence for sodium retention and the 7-day cumulative sodium balance was more negative during the high salt intake period (Figure 2). During control diet, there was a diurnal variation in the urinary excretion of sodium, chloride, and urine flow (Figure 2), which were all higher during the active phase. This diurnal rhythm was lost after 7 days of high salt feeding, such that the total daily excretion of sodium and chloride were evenly distributed across the day-night cycle.

Drinking behavior also became dysregulated after a week of high salt (Figure S7), and high salt–induced both polydipsia and polyuria (Figure S6) with mice entering positive cumulative water balance (Figure 2). Hematocrit, indicative of effective circulating volume, was unaffected by high salt feeding (Figure 2).

In a separate, cross-sectional study, high salt intake suppressed plasma aldosterone but only the 7- and 14-day measurements were different from control diet (Figure S8). At the mRNA level, high-salt diet reduced the expression of *slc9a3*, encoding NHE3 in the proximal tubule, and *scnn1a*, encoding the α-subunit of ENaC in the aldosterone-sensitive distal nephron. The expression of mRNA encoding the sodium-potassium-chloride cotransporter (NKCC2) and the sodium-chloride cotransporter (NCC) was unaffected by salt intake. There was a transient reduction in aquaporin-2 expression after 7 days of high salt intake (Figure S8).

### Pressure Natriuresis Response and Renal Hemodynamics

**Figure 3.**
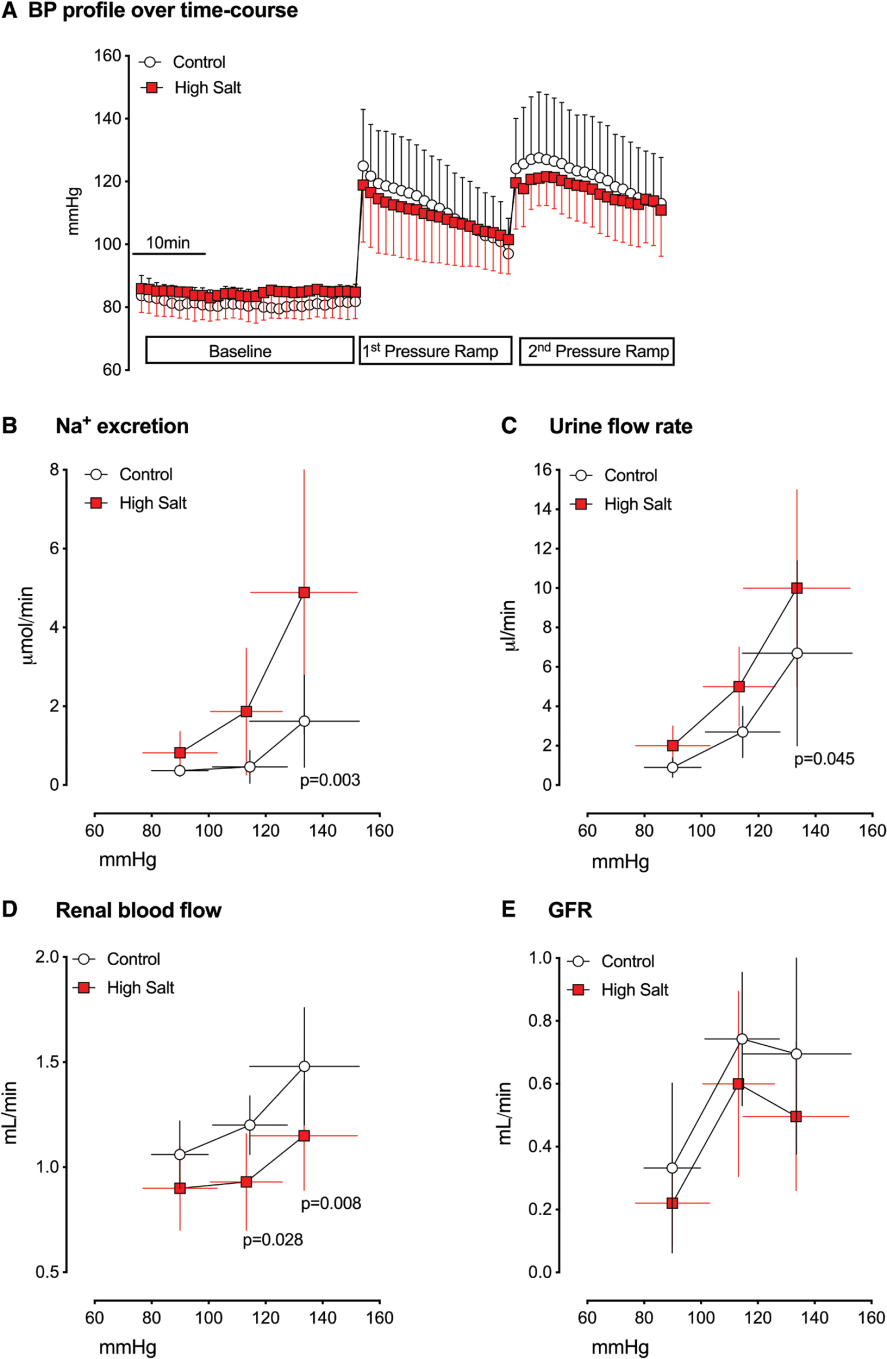
**The acute pressure natriuresis relationship in anesthetized male C57BL6/J mice fed either control diet (0.3% Na, open circles; n=9) or high sodium (3% Na; red squares; n=7) for 3 days.** Blood pressure (BP) was acutely raised over baseline by sequential ligation of the mesenteric and coeliac arteries (first pressure ramp) and the distal aorta (second pressure ramp). And urine collected via bladder cannula. **A**, The BP profile over the time course was not different between groups. **B**, Sodium excretion, (**C**) urine flow rate, (**D**) renal blood flow, (**F**) glomerular filtration rate (GFR). Data are group mean±SD and statistical analysis was by 2-way ANOVA. *P* values for planned comparisons between groups are given, made Holm-Sidak testing.

We hypothesized that the pressure natriuresis relationship, a key physiological regulator of BP, was attenuated in C57BL/6J mice fed high salt leading to the increase in BP. In anesthetized mice fed either control or high-salt diet for 3 days, arterial ligation was used to acutely increase BP (Figure 3A shows an exemplar trace), inducing a significant natriuresis and diuresis in both groups (ANOVA, main effect of BP: *P*<0.001). Contrary to our hypothesis, the high salt group displayed an enhanced natriuretic and diuretic (ANOVA, main effect of diet: *P*=0.002 & *P*=0.015, respectively) response to increasing BP. Renal blood flow and glomerular filtration rate were both significantly lower (ANOVA, main effect of diet: *P*<0.001 & *P*=0.037, respectively) in the mice fed high salt (Figure 3). The hemodynamic response to increasing BP was similar in both groups.

### Ex Vivo Vasomotor Function

We next asked whether intrinsic vasomotor function was altered by high salt intake. Using wire-myography, the maximal contractile response in mesenteric and renal arteries was no different between the high salt and control groups. In mesenteric arteries (Figure S9), phenylephrine-induced vasoconstriction, and vasodilation in response to acetylcholine and sodium nitroprusside were not influenced by high salt intake. In the renal artery, the sensitivity to phenylephrine was significantly increased after 7-days of high salt (logEC_50_ high salt 6.63+0.41 versus control diet 6.14+0.03; *P*=0.019, Figure S10).

### Sympathetic Activity and Catecholamines

**Figure 4.**
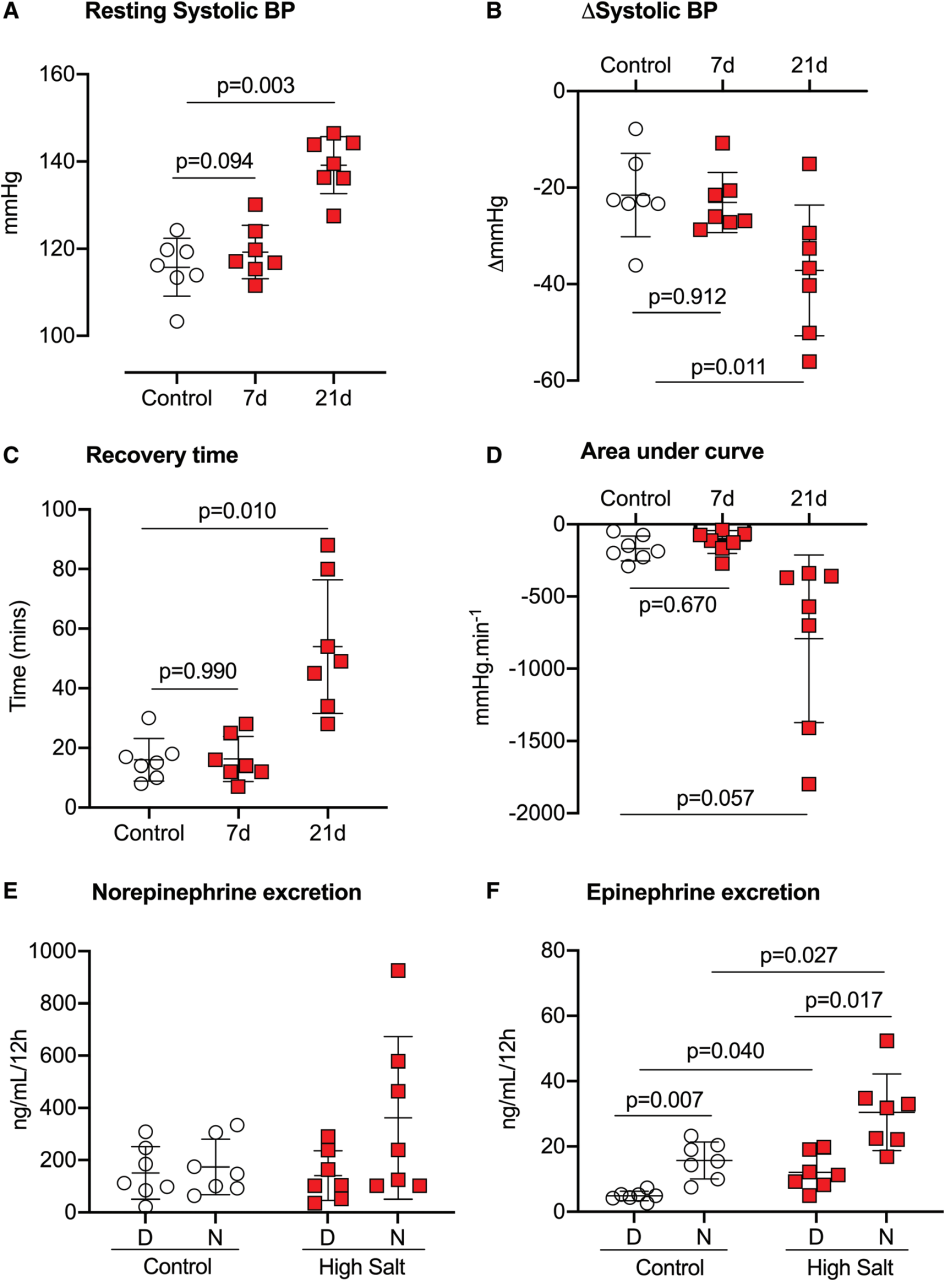
**The acute depressor effect of ganglionic blockade was used to index sympathetic nervous system activity in male C57BL6/J mice (n=7) fed control diet (0.3% Na, open circles) and then high-salt diet (3% Na; red squares) as indicated in the protocol (Figure S1).** **A**, resting systolic blood pressure (SBP). **B**, The maximum drop in SBP induced by hexamethonium (Δhexamethonium). **C**, the time taken for SBP to return to preinjection baseline. **D**, Area over the curve. Individual mouse data are shown with group mean±SD. Analysis was by repeated-measures 1-way ANOVA with *P* values shown for post-test comparisons to the control. In a separate group of mice (n=7), measurements of (**E**) urinary excretion of norepinephrine and (**F**) epinephrine during the day (D: inactive phase 7 am–7 pm) and night (N: active phase 7 pm–7 am) were made first on control diet (0.3% Na, open circles) and then after 7-d of high-salt diet (3% Na; red squares). Individual mice and group means±SD are shown. Analysis was by repeated-measures 2-way ANOVA, with main effects of diet and time of day. Planned post-test comparisons were performed by Holm-Sidak with *P* values indicated.

The acute depressor effect of the ganglionic blocker hexamethonium was used to index SNS activity on control diet and after 7 and 21 days of high-salt diet. Resting systolic BP was reduced significantly by hexamethonium (Figure 4, 1-sample *t* test: *P*<0.0001). During control diet, and after 7 days of high salt, systolic BP was reduced by ≈20 mm Hg, with a recovery time of ≈20 minutes. The area under the curve was not different between these 2 time points (Figure 4). After 21 days of high salt, the effect of hexamethonium was enhanced, reducing systolic BP by ≈40 mm Hg, with a recovery time of ≈50 minutes and significantly increased area under the curve.

Next, in another group of mice, we measured the urinary excretion of catecholamines first on control diet and again, starting on the seventh day of high salt intake. Norepinephrine spillover did not show clear diurnal variation (Figure 4E. ANOVA main effect of time of day: *P*=0.073), and excretion was unaffected after 7 days of high salt intake (ANOVA main effect of diet: *P*=0.255). Epinephrine excretion was higher during the night than during the day (Figure 4F. ANOVA main effect of time of day: *P*<0.0001), and was increased after 7 days of high salt in both active and inactive phases (ANOVA main effect of diet: *P*=0.004). Urine excretion of nitrite/nitrate, taken as an index of in vivo nitric oxide, was significantly reduced by high-salt diet (Figure S11).

## Discussion

Salt-sensitivity is an independent cardiovascular risk factor^12^ and, given habitually high salt intake in much of the world, presents an important health challenge. Mechanisms of salt-sensitivity are contentious with natriuretic dysfunction,^13^ vasodysfunction,^14^ and neurogenic paradigms^15^ dominating the debate. Our study finds that high-salt diet increases systolic BP by ≈10 mm Hg in commercially sourced, male C57BL6/J mice and that this salt-sensitivity is due activation of the sympathetic nervous system.

C57BL6/J and C57BL6/N mice are a widely used tool in biomedical research.^31^ In experimental hypertension, this strain serves as platform for pharmacological models, such as chronic ANG II (angiotensin II) infusion, and as the genetic background for informative transgenics (eg, International Mouse Phenotyping Consortium; www.mousephenotype.org). Their utility, outlined in The American Heart Association Scientific Statement on Animal Models of Hypertension,^18^ rests on the premise that the C57BL6 strain is intrinsically salt-resistant. The utility of continuous measurement of BP in nonrestrained animals by radio-telemetry is key. It allows a more nuanced interpretation of BP phenotypes than does the snap-shot provided by tail-cuff plethysmography. In head-to-head comparisons, telemetry demonstrated salt-sensitivity in C57BL6/J and N mice, whereas this important phenotype was obscured by tail-cuff.^27^ The advantages of direct BP measurement by radio-telemetry are widely acknowledged and The American Heart Association Council on High Blood Pressure Research recommends this approach when the scientific objective is to identify and quantify subtle forms of hypertension.^32^ Our study aligns with the overall conclusion: the BP response to salt cannot be assumed and radio-telemetry is sine qua non when ascribing salt-resistance or salt-sensitivity in experimental hypertension research.

A kidney-centered paradigm suggests that the rise in BP in response to high salt is due to a failure of pressure natriuresis, a physiological mechanism that acts to maintain extracellular fluid volume and buffer BP. In the current study, we observed that suppression of aldosterone in response to high salt occurred late after BP had plateaued. From our previous work, we hypothesized that aldosterone-sensitive sodium transporters were not downregulated rapidly at the initiation of high salt intake^33,34^ causing natriuretic dysfunction and sodium retention. This hypothesis was not supported: high-salt diet induced a rapid downregulation of αENaC mRNA, the rate-limiting step for functional ENaC formation, and no effects at the mRNA level on the aldosterone-sensitive transport proteins, NCC and NKCC2. Additionally, and in keeping with earlier work,^35,36^ we found that NHE3, a major proximal tubule sodium reabsorption pathway, was also downregulated. Importantly, functional downregulation of NHE3 is a major mechanism of pressure natriuresis,^16^ and this likely contributes to the increased natriuresis seen here. Overall, we found no evidence for sodium retention, and our data suggest that natriuretic dysfunction is not a major component to salt-sensitivity in C57BL6/J mice. We identified a subtle natriuretic abnormality in that diurnal variation of renal salt excretion was lost after 3 weeks of high salt intake. It is proposed that natriuretic dysfunction redistributes sodium excretion more evenly across the 24-hour cycle, necessitating a higher BP during the sleep phase to achieve sodium balance.^37,38^ This is important since loss of the normal diurnal variation in BP—nondipping—increases cardiovascular risk.^39^ However, based on our data, we conclude that that abnormal patterning of renal sodium excretion does not influence diurnal BP variation. Our study is consistent with previous work indicating that heart rate and cardiac output are the major determinants of a normally dipping BP.^40^

Studies in humans indicate that high salt intake initiates sodium retention and increased cardiac output but importantly the response is not greater in salt-sensitives than it is in salt-resistant controls.^41,42^ Instead, the salt-induced rise in BP is driven by a failure to appropriately vasodilate and reduce peripheral vascular resistance in salt-sensitive individuals and experimental models.^14^ We did not find intrinsic arterial abnormalities in nitric oxide-dependent or independent vasodilation, but longer-term studies indicate that high salt can lead to nitric oxide synthase uncoupling.^43^ Indeed, reduced urinary nitrite/nitrate excretion points to reduced global nitric oxide production in vivo, and circulating epinephrine is also likely elevated (discussed below). We also noted a modestly increased sensitivity to α-adrenergic activation, in the renal artery at least. Overall, it is reasonable to conclude that high-salt intake induces a proconstriction environment in *vivo*.

In healthy humans, high salt intake suppresses plasma and urine norepinephrine epinephrine.^44^ The salt-induced suppression was attenuated in salt-sensitive hypertensives.^44,45^ An important finding in our study was that high-salt diet did not suppress urinary norepinephrine excretion; urinary epinephrine spillover was significantly increased with high salt. Urinary catecholamine spillover reflects plasma concentrations over time,^46^ and our data suggest 2 things. First, that sympathetic activation is not suppressed by high salt intake, as would be expected in salt-resistance. Second, that adrenal epinephrine production is enhanced, as previously reported in salt-sensitive,^47^ but not salt-resistant^48^ rats. Coupled with the modest increase in intrinsic renal artery sensitivity to α_1_-adrenergic stimulation, enhanced circulating epinephrine and sustained sympathetic outflow^49^ might contribute to the salt-sensitive hypertension C57BL6/J mice.

Abnormal sympathetic activity during the high salt feeding phase of our telemetry experiment is also indicated by the exaggerated depressor response to hexamethonium, given here at a dose which eliminates renal sympathetic nerve activity.^50^ Measured only during the inactive phase, the response to hexamethonium likely underestimates the overall sympathetic contribution to BP in the stable phase of salt-sensitivity. This warrants further study but direct, longitudinal measurements of sympathetic activity by radio-telemetry are still an emerging technology.^51^ Nevertheless, our data strongly support a neurogenic component to salt-sensitivity in C57BL6/J mice that is not reliant on impaired natriuresis and sodium retention, as proposed by recent modeling.^52^

### Limitations

Of the several limitations in our study, we draw focus to three. First, we cannot unequivocally attribute the BP rise to increased dietary sodium chloride alone. Other constituents may vary between the diets and exert modifying effects on BP. For example, the high-salt diet was slightly lower in potassium (0.6% versus 0.7%) and estimated to have lower arginine content (≈0.4%, compared with ≈0.9%). The hypertensive effect of diets low in potassium is well-established^53^ and may make a modest contribution here. The arginine differential may be more significant since low molar supplements of nitrate or beetroot extracts confer protection against hypertension in the Dahl Salt-Sensitive rat.^54^

Second, our telemetry BP experiments were performed at an ambient temperature below thermoneutrality (30 °C). Both heart rate and BP increase as ambient temperature drops below a mouse’s thermoneutral zone^55^ and cold-stress is increasingly recognized as an important phenotypic modifier.^56^ Although the temperature effect is less pronounced during the active phase, when salt-sensitivity was most evident in our mice, it is possible that temperature-induced mild stress and increased salt intake combine to activate the sympathetic nervous system. Notably, salt-sensitive humans show an exaggerated tachycardia and cortisol response when exposed to acute mental stress, although norepinephrine levels are reduced compared with salt-resistant controls.^57^

Third, other studies, including ours, report the C57BL6/J background as salt-resistant.^22,23,25,26^ Phenotypic variation in the BP response to salt is also noted in the Dahl S rat, a classical model of salt-sensitivity.^58^ As highlighted by Rapp and Garrett,^58^ genetic divergence from a parent strain may contribute but other factors, particularly around experimental design and dietary salt load make significant contributions. The statistical framework is also a factor: experiments designed to compare between genotypes can lack power to discriminate a within-group effect and may identify subtle increases in BP as a nonsignificant trend.^23^

### Perspectives

Hypertension research is reliant on mouse models and the C57BL6J mouse is commonly used. Understanding whether or not a particular rodent strain is salt-sensitive or salt-resistant is desirable as it informs the rational design of experimental protocols and the interpretation of hemodynamic data. So are C57BL6J mice salt-sensitive or salt-resistant? In the absence of an accepted industry standard quantitative definition of salt-sensitivity, thresholds are still arbitrary.^18^ Certainly we show a sustained ≈10 mm Hg BP rise with a ≈10-fold increase in dietary salt intake but this effect is perhaps small compared with established models of salt-sensitivity. The binary categorization of resistant or sensitive does not accurately reflect the underlying biology of is a continuous trait but is experimentally convenient and indeed useful. Our study does not alter the applicability of the C57BL6J as a work-horse for hypertension research but provides important information for researchers to consider when designing and interpreting their studies.

## Acknowledgments

We thank David Binnie for supporting animal welfare, randomization, and blinding during this study.

## Sources of Funding

This work was funded by PhD studentships from The University of Edinburgh/Zhejiang and British Heart Foundation (FS/16/54/32730) and by research grants from the British Heart Foundation (PG/16/98/32568) and Kidney Research UK (IN001/20170302).

## Disclosures

None.

## Supplementary Material



## Novelty and Significance

### What Is New?

- This study uses radio-telemetry to show that high-salt diet increases blood pressure (BP) in male C57BL6J mice sourced from a major commercial supplier.
- Salt-sensitive BP was not caused by renal sodium retention and the pressure natriuresis relationship was enhanced, not attenuated.
- High BP was neurogenic and potentially vascular, due to increased sympathetic activity, elevated levels of epinephrine, and enhanced renal arterial vascular sensitivity to α-adrenoreceptor stimulation.

### What Is Relevant?

- C57BL6 mice are widely used in hypertension research and identified in the most recent American Heart Association Animal Models of Hypertension: A Scientific Statement as having salt-resistant BP.
- This study suggests salt-sensitivity or salt-resistance of inbred rodents cannot be assumed, highlighting the importance to laboratories of profiling their background strain using radio-telemetry when BP is a major study end point.
- Mechanistically, the study suggests that salt-sensitivity can develop without sodium retention and further indicates that diurnal variation in BP is not influenced by diurnal variation in salt excretion.

### Summary

We found salt-sensitivity in commercially sourced male C57BL6/J mice, reflecting sympathetic overactivity and not sodium retention or attenuation of the acute pressure natriuresis response.

## References

[1] Carrillo-Larco RM Bernabe-Ortiz A Sodium and salt consumption in Latin America and the Caribbean: a systematic-review and meta-analysis of population-based studies and surveys. Nutrients. 2020;12:556 10.3390/nu12020556 PMC7071427 32093337

[2] Neupane D Rijal A Henry ME Kallestrup P Koirala B Mclachlan CS Ghimire K Zhao D Sharma S Pokharel Y Mean dietary salt intake in Nepal: a population survey with 24-hour urine collections. J Clin Hypertens (Greenwich). 2020;22:273–279. doi: 10.1111/jch.13813 31967732 10.1111/jch.13813 PMC8029777

[3] Webb M Fahimi S Singh GM Khatibzadeh S Micha R Powles J Mozaffarian D Cost effectiveness of a government supported policy strategy to decrease sodium intake: global analysis across 183 nations. BMJ. 2017;356:i6699 doi: 10.1136/bmj.i6699 28073749 10.1136/bmj.i6699 PMC5225236

[4] Whelton PK Appel LJ Sacco RL Anderson CA Antman EM Campbell N Dunbar SB Frohlich ED Hall JE Jessup M Sodium, blood pressure, and cardiovascular disease: further evidence supporting the American Heart Association sodium reduction recommendations. Circulation. 2012;126:2880–2889. doi: 10.1161/CIR.0b013e318279acbf 23124030 10.1161/CIR.0b013e318279acbf

[5] Sigaux J Semerano L Favre G Bessis N Boissier MC Salt, inflammatory joint disease, and autoimmunity. Joint Bone Spine. 2018;85:411–416. doi: 10.1016/j.jbspin.2017.06.003 28652101 10.1016/j.jbspin.2017.06.003

[6] Kang M Kang E Ryu H Hong Y Han SS Park SK Hyun YY Sung SA Kim SW Yoo TH Measured sodium excretion is associated with CKD progression: results from the KNOW-CKD study. Nephrol Dial Transplant. 2020 gfaa107 doi: 10.1093/ndt/gfaa107 32582942 10.1093/ndt/gfaa107

[7] Faraco G Hochrainer K Segarra SG Schaeffer S Santisteban MM Menon A Jiang H Holtzman DM Anrather J Iadecola C Dietary salt promotes cognitive impairment through tau phosphorylation. Nature. 2019;574:686–690. doi: 10.1038/s41586-019-1688-z 31645758 10.1038/s41586-019-1688-z PMC7380655

[8] Fang X Wei J He X An P Wang H Jiang L Shao D Liang H Li Y Wang F Landscape of dietary factors associated with risk of gastric cancer: a systematic review and dose-response meta-analysis of prospective cohort studies. Eur J Cancer. 2015;51:2820–2832. doi: 10.1016/j.ejca.2015.09.010 26589974 10.1016/j.ejca.2015.09.010

[9] Strom BL Yaktine AL Oria M, eds. Sodium Intake in Populations: Assessment of Evidence. 2013 National Academies Press 24851297

[10] Franco V Oparil S Salt sensitivity, a determinant of blood pressure, cardiovascular disease and survival. J Am Coll Nutr. 2006;25 3 suppl 247S–255S. doi: 10.1080/07315724.2006.10719574 16772636 10.1080/07315724.2006.10719574

[11] Weinberger MH Fineberg NS Fineberg SE Weinberger M Salt sensitivity, pulse pressure, and death in normal and hypertensive humans. Hypertension. 2001;37 2 pt 2 429–432. doi: 10.1161/01.hyp.37.2.429 11230313 10.1161/01.hyp.37.2.429

[12] Morimoto A Uzu T Fujii T Nishimura M Kuroda S Nakamura S Inenaga T Kimura G Sodium sensitivity and cardiovascular events in patients with essential hypertension. Lancet. 1997;350:1734–1737. doi: 10.1016/S0140-6736(97)05189-1 9413464 10.1016/S0140-6736(97)05189-1

[13] Hall JE Renal dysfunction, rather than nonrenal vascular dysfunction, mediates salt-induced hypertension. Circulation. 2016;133:894–906. doi: 10.1161/CIRCULATIONAHA.115.018526 26927007 10.1161/CIRCULATIONAHA.115.018526 PMC5009905

[14] Morris RC Jr Schmidlin O Sebastian A Tanaka M Kurtz TW Vasodysfunction that involves renal vasodysfunction, not abnormally increased renal retention of sodium, accounts for the initiation of salt-induced hypertension. Circulation. 2016;133:881–893. doi: 10.1161/CIRCULATIONAHA.115.017923 26927006 10.1161/CIRCULATIONAHA.115.017923 PMC4778403

[15] Averina VA Othmer HG Fink GD Osborn JW A mathematical model of salt-sensitive hypertension: the neurogenic hypothesis. J Physiol. 2015;593:3065–3075. doi: 10.1113/jphysiol.2014.278317 26173827 10.1113/jphysiol.2014.278317 PMC4532527

[16] Ivy JR Bailey MA Pressure natriuresis and the renal control of arterial blood pressure. J Physiol. 2014;592:3955–3967. doi: 10.1113/jphysiol.2014.271676 25107929 10.1113/jphysiol.2014.271676 PMC4198007

[17] Bie P Evans RG Normotension, hypertension and body fluid regulation: brain and kidney. Acta Physiol (Oxf). 2017;219:288–304. doi: 10.1111/apha.12718 27214656 10.1111/apha.12718

[18] Lerman LO Kurtz TW Touyz RM Ellison DH Chade AR Crowley SD Mattson DL Mullins JJ Osborn J Eirin A Animal models of hypertension: a scientific statement from the American Heart Association. Hypertension. 2019;73:e87–e120. doi: 10.1161/HYP.0000000000000090 30866654 10.1161/HYP.0000000000000090 PMC6740245

[19] Mullins LJ Bailey MA Mullins JJ Hypertension, kidney, and transgenics: a fresh perspective. Physiol Rev. 2006;86:709–746. doi: 10.1152/physrev.00016.2005 16601272 10.1152/physrev.00016.2005

[20] Czopek A Moorhouse R Guyonnet L Farrah T Lenoir O Owen E van Bragt J Costello HM Menolascina F Baudrie V A novel role for myeloid endothelin-B receptors in hypertension. Eur Heart J. 2019;40:768–784. doi: 10.1093/eurheartj/ehy881 30657897 10.1093/eurheartj/ehy881 PMC6396028

[21] Dasinger JH Alsheikh AJ Abais-Battad JM Pan X Fehrenbach DJ Lund H Roberts ML Cowley AW Jr Kidambi S Kotchen TA Epigenetic modifications in T cells: the role of DNA methylation in salt-sensitive hypertension. Hypertension. 2020;75:372–382. doi: 10.1161/HYPERTENSIONAHA.119.13716 31838911 10.1161/HYPERTENSIONAHA.119.13716 PMC7058976

[22] Evans LC Ivy JR Wyrwoll C McNairn JA Menzies RI Christensen TH Al-Dujaili EA Kenyon CJ Mullins JJ Seckl JR Conditional deletion of Hsd11b2 in the brain causes salt appetite and hypertension. Circulation. 2016;133:1360–1370. doi: 10.1161/CIRCULATIONAHA.115.019341 26951843 10.1161/CIRCULATIONAHA.115.019341 PMC4819772

[23] Wu J Agbor LN Fang S Mukohda M Nair AR Nakagawa P Sharma A Morgan DA Grobe JL Rahmouni K Failure to vasodilate in response to salt loading blunts renal blood flow and causes salt-sensitive hypertension. Cardiovasc Res. 2020 cvaa147 doi: 10.1093/cvr/cvaa147 10.1093/cvr/cvaa147 PMC7797211 32428209

[24] Lum C Shesely EG Potter DL Beierwaltes WH Cardiovascular and renal phenotype in mice with one or two renin genes. Hypertension. 2004;43:79–86. doi: 10.1161/01.HYP.0000107401.72456.50 14662650 10.1161/01.HYP.0000107401.72456.50

[25] Bailey MA Craigie E Livingstone DEW Kotelevtsev YV Al-Dujaili EAS Kenyon CJ Mullins JJ Hsd11b2 haploinsufficiency in mice causes salt sensitivity of blood pressure. Hypertension. 2011;57:515–520. doi: 10.1161/HYPERTENSIONAHA.110.163782 21282561 10.1161/HYPERTENSIONAHA.110.163782 PMC4830399

[26] Hartner A Cordasic N Klanke B Veelken R Hilgers KF Strain differences in the development of hypertension and glomerular lesions induced by deoxycorticosterone acetate salt in mice. Nephrol Dial Transplant. 2003;18:1999–2004. doi: 10.1093/ndt/gfg299 13679473 10.1093/ndt/gfg299

[27] Combe R Mudgett J El Fertak L Champy MF Ayme-Dietrich E Petit-Demoulière B Sorg T Herault Y Madwed JB Monassier L How does circadian rhythm impact salt sensitivity of blood pressure in mice? A study in two close C57Bl/6 substrains. PLoS One. 2016;11:e0153472 doi: 10.1371/journal.pone.0153472 27088730 10.1371/journal.pone.0153472 PMC4835052

[28] Helkamaa T Männistö PT Rauhala P Cheng ZJ Finckenberg P Huotari M Gogos JA Karayiorgou M Mervaala EM Resistance to salt-induced hypertension in catechol-O-methyltransferase-gene-disrupted mice. J Hypertens. 2003;21:2365–2374. doi: 10.1097/00004872-200312000-00026 14654758 10.1097/00004872-200312000-00026

[29] Ivy JR Oosthuyzen W Peltz TS Howarth AR Hunter RW Dhaun N Al-Dujaili EA Webb DJ Dear JW Flatman PW Glucocorticoids induce nondipping blood pressure by activating the thiazide-sensitive cotransporter. Hypertension. 2016;67:1029–1037. doi: 10.1161/HYPERTENSIONAHA.115.06977 26953322 10.1161/HYPERTENSIONAHA.115.06977 PMC4905621

[30] Kimura G Dohi Y Fukuda M Salt sensitivity and circadian rhythm of blood pressure: the keys to connect CKD with cardiovascular events. Hypertens Res. 2010;33:515–520. doi: 10.1038/hr.2010.47 20379191 10.1038/hr.2010.47

[31] Festing MF Evidence should trump intuition by preferring inbred strains to outbred stocks in preclinical research. ILAR J. 2014;55:399–404. doi: 10.1093/ilar/ilu036 25541542 10.1093/ilar/ilu036

[32] Kurtz TW Griffin KA Bidani AK Davisson RL Hall JE; Subcommittee of Professional and Public Education of the American Heart Association. Recommendations for blood pressure measurement in humans and experimental animals. Part 2: blood pressure measurement in experimental animals: a statement for professionals from the subcommittee of professional and public education of the American Heart Association council on high blood pressure research. Hypertension. 2005;45:299–310. doi: 10.1161/01.HYP.0000150857.39919.cb 15611363 10.1161/01.HYP.0000150857.39919.cb

[33] Craigie E Evans LC Mullins JJ Bailey MA Failure to downregulate the epithelial sodium channel causes salt sensitivity in Hsd11b2 heterozygote mice. Hypertension. 2012;60:684–690. doi: 10.1161/HYPERTENSIONAHA.112.196410 22777941 10.1161/HYPERTENSIONAHA.112.196410 PMC3428628

[34] Ivy JR Evans LC Moorhouse R Richardson RV Al-Dujaili EAS Flatman PW Kenyon CJ Chapman KE Bailey MA Renal and blood pressure response to a high-salt diet in mice with reduced global expression of the glucocorticoid receptor. Front Physiol. 2018;9:848 doi: 10.3389/fphys.2018.00848 30038578 10.3389/fphys.2018.00848 PMC6046455

[35] Yang LE Sandberg MB Can AD Pihakaski-Maunsbach K McDonough AA Effects of dietary salt on renal Na+ transporter subcellular distribution, abundance, and phosphorylation status. Am J Physiol Renal Physiol. 2008;295:F1003–F1016. doi: 10.1152/ajprenal.90235.2008 18653479 10.1152/ajprenal.90235.2008 PMC2576159

[36] Udwan K Abed A Roth I Dizin E Maillard M Bettoni C Loffing J Wagner CA Edwards A Feraille E Dietary sodium induces a redistribution of the tubular metabolic workload. J Physiol. 2017;595:6905–6922. doi: 10.1113/JP274927 28940314 10.1113/JP274927 PMC5685825

[37] Fukuda M Goto N Kimura G Hypothesis on renal mechanism of non-dipper pattern of circadian blood pressure rhythm. Med Hypotheses. 2006;67:802–806. doi: 10.1016/j.mehy.2006.04.024 16759814 10.1016/j.mehy.2006.04.024

[38] Bankir L Bochud M Maillard M Bovet P Gabriel A Burnier M Nighttime blood pressure and nocturnal dipping are associated with daytime urinary sodium excretion in African subjects. Hypertension. 2008;51:891–898. doi: 10.1161/HYPERTENSIONAHA.107.105510 18316653 10.1161/HYPERTENSIONAHA.107.105510

[39] Ivy JR Bailey MA Non-dipping blood pressure: predictive or reactive failure of renal sodium handling? Physiology. 2020 doi: 10.1152/physiol.00024.2020 10.1152/physiol.00024.2020 33325814

[40] Kurtz TW Lujan HL DiCarlo SE The 24 h pattern of arterial pressure in mice is determined mainly by heart rate-driven variation in cardiac output. Physiol Rep. 2014;2:e12223 25428952 10.14814/phy2.12223 PMC4255824

[41] Schmidlin O Forman A Leone A Sebastian A Morris RC Jr. Salt sensitivity in blacks: evidence that the initial pressor effect of NaCl involves inhibition of vasodilatation by asymmetrical dimethylarginine. Hypertension. 2011;58:380–385. doi: 10.1161/HYPERTENSIONAHA.111.170175 21788605 10.1161/HYPERTENSIONAHA.111.170175

[42] Schmidlin O Sebastian AF Morris RC Jr. What initiates the pressor effect of salt in salt-sensitive humans? Observations in normotensive blacks. Hypertension. 2007;49:1032–1039. doi: 10.1161/HYPERTENSIONAHA.106.084640 17372035 10.1161/HYPERTENSIONAHA.106.084640 PMC2765792

[43] Nurkiewicz TR Wu G Li P Boegehold MA Decreased arteriolar tetrahydrobiopterin is linked to superoxide generation from nitric oxide synthase in mice fed high salt. Microcirculation. 2010;17:147–157. doi: 10.1111/j.1549-8719.2009.00014.x 20163541 10.1111/j.1549-8719.2009.00014.x PMC3402363

[44] Campese VM Romoff MS Levitan D Saglikes Y Friedler RM Massry SG Abnormal relationship between sodium intake and sympathetic nervous system activity in salt-sensitive patients with essential hypertension. Kidney Int. 1982;21:371–378. doi: 10.1038/ki.1982.32 7069999 10.1038/ki.1982.32

[45] Miyajima E Yamada Y Reduced sympathetic inhibition in salt-sensitive Japanese young adults. Am J Hypertens. 1999;12 12pt 1-2 1195–1200. doi: 10.1016/s0895-7061(99)00122-3 10619582 10.1016/s0895-7061(99)00122-3

[46] Baines AD Effects of salt intake and renal denervation on catecholamine catabolism and excretion. Kidney Int. 1982;21:316–322. doi: 10.1038/ki.1982.24 7069996 10.1038/ki.1982.24

[47] Saavedra JM Del Carmine R McCarty R Guicheney P Weise V Iwai J Increased adrenal catecholamines in salt-sensitive genetically hypertensive Dahl rats. Am J Physiol. 1983;245 5pt 1 H762–H766. doi: 10.1152/ajpheart.1983.245.5.H762 6139026 10.1152/ajpheart.1983.245.5.H762

[48] Inglis GC Kenyon CJ Hannah JA Connell JM Ball SG Does dopamine regulate aldosterone secretion in the rat? Clin Sci (Lond). 1987;73:93–97. doi: 10.1042/cs0730093 3608380 10.1042/cs0730093

[49] Malpas SC Sympathetic nervous system overactivity and its role in the development of cardiovascular disease. Physiol Rev. 2010;90:513–557. doi: 10.1152/physrev.00007.2009 20393193 10.1152/physrev.00007.2009

[50] Hamza SM Hall JE Direct recording of renal sympathetic nerve activity in unrestrained, conscious mice. Hypertension. 2012;60:856–864. doi: 10.1161/HYPERTENSIONAHA.111.186577 22851730 10.1161/HYPERTENSIONAHA.111.186577 PMC3864832

[51] Hart EC Head GA Carter JR Wallin BG May CN Hamza SM Hall JE Charkoudian N Osborn JW Recording sympathetic nerve activity in conscious humans and other mammals: guidelines and the road to standardization. Am J Physiol Heart Circ Physiol. 2017;312:H1031–H1051. doi: 10.1152/ajpheart.00703.2016 28364017 10.1152/ajpheart.00703.2016 PMC6146303

[52] Averina VA Othmer HG Fink GD Osborn JW A new conceptual paradigm for the haemodynamics of salt-sensitive hypertension: a mathematical modelling approach. J Physiol. 2012;590:5975–5992. doi: 10.1113/jphysiol.2012.228619 22890716 10.1113/jphysiol.2012.228619 PMC3530111

[53] Kanbay M Bayram Y Solak Y Sanders PW Dietary potassium: a key mediator of the cardiovascular response to dietary sodium chloride. J Am Soc Hypertens. 2013;7:395–400. doi: 10.1016/j.jash.2013.04.009 23735420 10.1016/j.jash.2013.04.009 PMC4083820

[54] Morris RC Jr Pravenec M Šilhavý J DiCarlo SE Kurtz TW Small amounts of inorganic nitrate or beetroot provide substantial protection from salt-induced increases in blood pressure. Hypertension. 2019;73:1042–1048. doi: 10.1161/HYPERTENSIONAHA.118.12234 30917704 10.1161/HYPERTENSIONAHA.118.12234 PMC6458074

[55] Williams TD Chambers JB Henderson RP Rashotte ME Overton JM Cardiovascular responses to caloric restriction and thermoneutrality in C57BL/6J mice. Am J Physiol Regul Integr Comp Physiol. 2002;282:R1459–R1467. doi: 10.1152/ajpregu.00612.2001 11959690 10.1152/ajpregu.00612.2001

[56] Maloney SK Fuller A Mitchell D Gordon C Overton JM Translating animal model research: does it matter that our rodents are cold? Physiology (Bethesda). 2014;29:413–420. doi: 10.1152/physiol.00029.2014 25362635 10.1152/physiol.00029.2014

[57] Weber CS Thayer JF Rudat M Sharma AM Perschel FH Buchholz K Deter HC Salt-sensitive men show reduced heart rate variability, lower norepinephrine and enhanced cortisol during mental stress. J Hum Hypertens. 2008;22:423–431. doi: 10.1038/jhh.2008.11 18337758 10.1038/jhh.2008.11

[58] Rapp JP Garrett MR Will the real Dahl S rat please stand up? Am J Physiol Renal Physiol. 2019;317:F1231–F1240. doi: 10.1152/ajprenal.00359.2019 31545925 10.1152/ajprenal.00359.2019 PMC6879929

