# Supplementary figures and images for: Activation of the Sympathetic Nervous System Promotes Blood Pressure Salt-Sensitivity in C57BL6/J Mice
Source: Hypertension. 2020 Nov 16;77(1):158–68. doi: 10.1161/HYPERTENSIONAHA.120.16186 (PMC7720873; doi:10.1161/HYPERTENSIONAHA.120.16186)

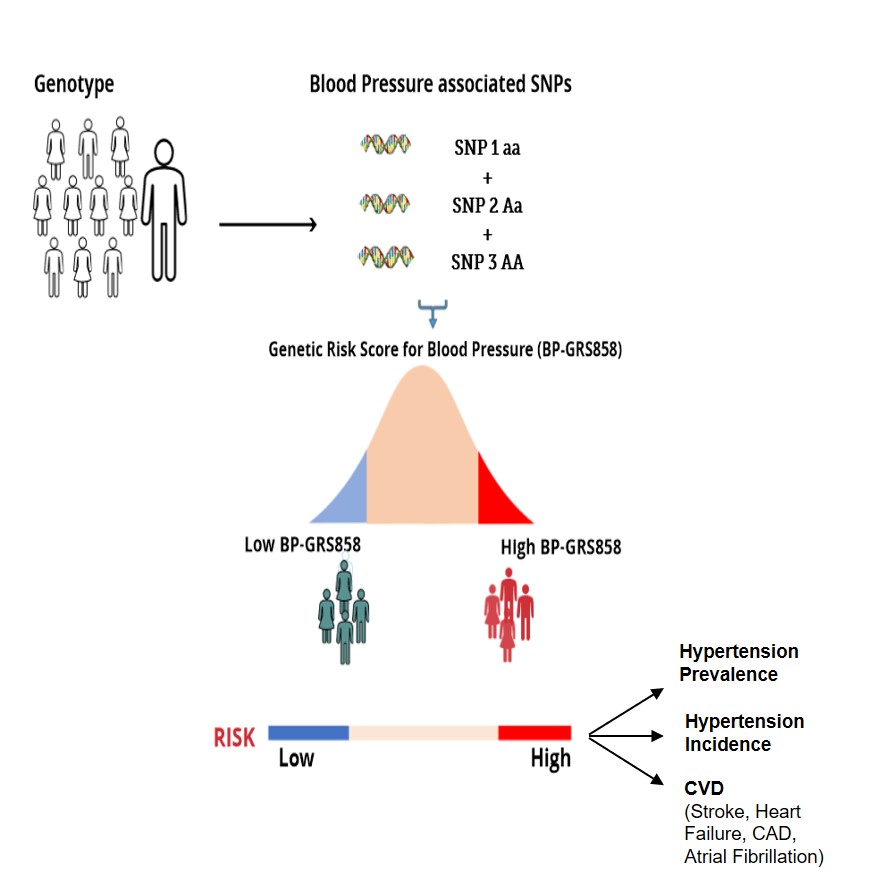

Supplement: Supplementary file 1 [file hyp-77-158-s001.jpg]
