# Supplementary material for: Activation of the Sympathetic Nervous System Promotes Blood Pressure Salt-Sensitivity in C57BL6/J Mice
Source: Hypertension. 2020 Nov 16;77(1):158–68. doi: 10.1161/HYPERTENSIONAHA.120.16186 (PMC7720873; doi:10.1161/HYPERTENSIONAHA.120.16186)
Supplement: Supplementary file 2 [file hyp-77-158-s002.pdf]

Online Supplement - Clinical evaluation of the polygenetic background of blood pressure in the population-based setting

Alice Giontella[1,2], Marketa Sjögren[2], Luca A. Lotta[3], John D. Overton[3], Aris Baras[3], Regeneron Genetics Center[3§], Pietro Minuz[1], Cristiano Fava[1,2*], Olle Melander[2,4*]

1. Department of Medicine, University of Verona, Verona, Italy

2. Department of Clinical Sciences, Clinical Research Center, Lund University, Malmö, Sweden

3. Regeneron Genetics Center, Tarrytown, NY, USA. §Author list is provided in the supplementary material

4. Department of Emergency and Internal Medicine, Skåne University Hospital, Malmö, Sweden

Short Title: Polygenetic background of blood pressure

Corresponding Author:

Cristiano Fava

University of Verona, Department of Medicine

"General Medicine and Hypertension" Unit

AOUI - Hospital “Policlinico G.B. Rossi”

P.le LA Scuro 10

37134 Verona, Italy

Phone: +39 045 8124732

e-mail: cristiano.fava@univr.it

cristiano.fava@med.lu.se

*These authors contributed equally to the work

Supplementary material

Regeneron Genetics Center Banner Author List and Contribution Statements
All authors/contributors are listed in alphabetical order.
RGC Management and Leadership Team
Goncalo Abecasis, Ph.D., Aris Baras, M.D., Michael Cantor, M.D., Giovanni Coppola, M.D., Aris Economides, Ph.D., Luca A. Lotta, M.D., Ph.D., John D. Overton, Ph.D., Jeffrey G. Reid, Ph.D., Alan Shuldiner, M.D.
Contribution: All authors contributed to securing funding, study design and oversight. All authors reviewed the final version of the manuscript.
Sequencing and Lab Operations
Christina Beechert, Caitlin Forsythe, M.S., Erin D. Fuller, Zhenhua Gu, M.S., Michael Lattari, Alexander Lopez, M.S., John D. Overton, Ph.D., Thomas D. Schleicher, M.S., Maria Sotiropoulos Padilla, M.S., Karina Toledo, Louis Widom, Sarah E. Wolf, M.S., Manasi Pradhan, M.S., Kia Manoochehri, Ricardo H. Ulloa.
Contribution: C.B., C.F., K.T., A.L., and J.D.O. performed and are responsible for sample genotyping. C.B, C.F., E.D.F., M.L., M.S.P., K.T., L.W., S.E.W., A.L., and J.D.O. performed and are responsible for exome sequencing. T.D.S., Z.G., A.L., and J.D.O. conceived and are responsible for laboratory automation. M.P., K.M., R.U., and J.D.O are responsible for sample tracking and the library information management system.
Genome Informatics
Xiaodong Bai, Ph.D., Suganthi Balasubramanian, Ph.D., Leland Barnard, Ph.D., Andrew Blumenfeld, Gisu Eom, Lukas Habegger, Ph.D., Young Hahn, Alicia Hawes, B.S., Shareef Khalid, Jeffrey G. Reid, Ph.D., Evan K. Maxwell, Ph.D., William Salerno, Ph.D., Jeffrey C. Staples, Ph.D., Ashish Yadav, M.S.
Contribution: X.B., A.H., W.S. and J.G.R. performed and are responsible for analysis needed to produce exome and genotype data. G.E., Y.H., and J.G.R. provided compute infrastructure development and operational support. S.K., S.B., and J.G.R. provide variant and gene annotations and their functional interpretation of variants. E.M., L.B., J.S., A.B., A.Y., L.H., J.G.R. conceived and are responsible for creating, developing, and deploying analysis platforms and computational methods for analyzing genomic data.
Research Program Management
Marcus B. Jones, Ph.D., Lyndon J. Mitnaul, Ph.D.
Contribution: All authors contributed to the management and coordination of all research activities, planning and execution. All authors contributed to the review process for the final version of the manuscript.

Supplementary Methods

Cardiovascular events in MDC

Coronary artery diseases included fatal or nonfatal myocardial infarction, death from ischemic heart disease, and the treatment procedures coronary artery bypass graft surgery (CABG) and percutaneous coronary intervention (PCI), whichever of these events came first. Death due to ischemic heart disease was defined on the basis of the International Classification of Diseases ninth and tenth revisions (ICD-9 and ICD-10) codes 412 and 414 (ICD-9) or I22–I23 and I25 (ICD-10). Further on, myocardial infarction was defined by the codes 410 and I21 (ICD-9 and ICD-10, respectively). CABG was identified from national Swedish classification systems of surgical procedures, the KKÅ system from 1963 until 1989 and the Op6 system since then. CABG was defined as a procedure code of 3065, 3066, 3068,3080, 3092, 3105, 3127, 3158 (Op6) or FN (KKÅ97). The definition of PCI was made on the basis of the operation

codes FNG05 and FNG02. Stroke (fatal or nonfatal) was defined using codes 430, 431, 434 and 436 (ICD-9) and I60, I61, I63 and I64 (ICD-10).

Genotyping

Genotype was performed by Regeneron Genetic Centre using an Illumina GWAS chip (*GSA array v1*) according to manufacturer's standard protocols. Imputation was performed in 3 10k-sample batches (n=9,299) for third batch), phasing to reference panel "hr1.1.2016" for European population (software used: eagle v2.3). Before imputation, autosomic SNPs with Minor Allele frequency (MAF) greater than 1%, with less than 1% missingness, with a Handy-Weinberg equilibrium (HWE) less than $1xe^{-15}$ passed the quality control, resulting in a total number of 39,127,700 markers. Individuals with more than 10% of missing genotype data as well as blacklisted and duplicated ones were excluded.

Genetic Risk Score

A weighted score was computed with PLINK version 1.90b5.2 (*--score*) using effect alleles and weights for European Ancestry as reported in the two latest GWAS meta-analysis [1,2], for both SBP and DBP associated variants. We first compute two trait-specific GRS that were averaged to obtain blood pressure GRS_{858} including 858 variants. We derived a systolic blood pressure (SBP) GRS including 474 independent variants and a diastolic blood pressure (DBP) score including 384 variants (Tables **S1, S2**). Twenty-eight SNPs were included in both scores because associated to both traits. Independency among variants was tested using pairwise linkage disequilibrium (LD) statistics. LD matrix was computed using *PLINK version 1.90b5.2* (--ld) [3] and a threshold of r^2=0.1 have been set. Pairs of SNPs that were above threshold of r^2, and considered not independent, we retained the one with lowest reported p-value. For those variants that weren't in our data, we search for proxies in LD (r^2>0.8) using web-based application LDLink 3.7.2 (module: LDproxy, available at *https://ldlink.nci.nih.gov/?tab=ldproxy*) provided by National Cancer Institute [4].

Supplementary Results

The general characteristics of the individuals that were genotyped are shown **in table S3.**

Descriptive statistics of BP-GRS$_{858}$

Descriptive statistics of the GRS$_{858}$ are presented in table **S4** and the distribution of the score among the two cohorts is shown in **figures S1 and S2.**

Association of BP-GRS$_{858}$ with blood pressure traits

The association between BP-GRS$_{858}$ and blood pressure traits are shown in table**S5 for the MDC and table S6 for the MPP.** Two models were shown: one adjusted for age and sex and body mass index (model A) and one mutually adjusted with the old score BP-GRS$_{29}$ (model B)**.**

Bibliography

1. Evangelou E, Warren HR, Mosen-Ansorena D, et al. Genetic analysis of over 1 million people identifies 535 new loci associated with blood pressure traits. *Nat Genet*. 2018;50(10):1412-1425. doi:10.1038/s41588-018-0205-x

2. Giri A, Hellwege JN, Keaton JM, et al. Trans-ethnic association study of blood pressure determinants in over 750,000 individuals. *Nat Genet*. 2019;51(1):51-62. doi:10.1038/s41588-018-0303-9

3. Chang CC, Chow CC, Tellier LC, Vattikuti S, Purcell SM, Lee JJ. Second-generation PLINK: rising to the challenge of larger and richer datasets. *Gigascience*. 2015;4(1):7. doi:10.1186/s13742-015-0047-8

4. Machiela MJ, Chanock SJ. LDlink: a web-based application for exploring population-specific haplotype structure and linking correlated alleles of possible functional variants. *Bioinformatics*. 2015;31(21):3555-3557. doi:10.1093/bioinformatics/btv402

Supplementary Tables

Table S1 List of SNPs with weights and effect allele used to compute the SBP-GRS474

rsID	Chr	BP	MAF*	Minor Allele	Effect allele	Beta§	proxy	lead snps
rs1014988	1	1680219	0.4868	G	G	0.204		
rs260508	1	2187085	0.4106	G	T	0.149		
rs2252865	1	8422676	0.3486	T	T	0.187		
rs1815614	1	9441838	0.4259	G	A	0.241		
rs880315	1	10796866	0.3498	C	C	0.508		
rs17367504	1	11862778	0.1507	G	A	0.897		
rs3820068	1	15798197	0.2183	G	A	0.294		
rs2807337	1	22577371	0.37	T	T	0.175		
rs79598313	1	27284913	0.03077	T	T	0.593		
rs3737801	1	27960832	0.07512	G	C	0.298		
rs6689862	1	28837443	0.09473	C	C	0.327		
rs12569027	1	38449163	0.4696	T	T	0.196		
rs11210029	1	41865293	0.3893	G	G	0.148		
rs7515635	1	42408070	0.4923	C	T	0.251		
rs839755	1	43856410	0.3625	C	C	0.227		
rs11579440	1	49052423	0.1385	C	T	0.212		
rs12125786	1	56616712	0.3124	G	C	0.229		
rs7547570	1	67008495	0.3946	A	A	0.168		
rs10923038	1	88651771	0.3844	C	A	0.162		
rs10922502	1	89360158	0.347	G	G	0.286		
rs7514579	1	94051350	0.2234	C	A	0.197		
rs17396055	1	94730954	0.3254	A	G	0.173		

rs3790604	1	1.13E+08	0.08843	A	A	0.635		
rs76719272	1	1.56E+08	0.134	T	C	0.239		
rs2179490	1	1.69E+08	0.4216	A	G	0.231		
rs1043069	1	1.81E+08	0.3809	G	T	0.2		
rs4651224	1	1.85E+08	0.4515	T	T	0.194		
rs12042924	1	1.97E+08	0.4898	C	C	0.162		
rs33996239	1	2.03E+08	0.0541	T	C	0.354		
rs7550273	1	2.08E+08	0.4391	C	G	0.194		
rs7555285	1	2.10E+08	0.2343	G	C	0.196		
rs2494184	1	2.10E+08	0.4221	T	C	0.169		
rs12088448	1	2.19E+08	0.3525	C	C	0.163		
rs1415293	1	2.20E+08	0.3126	T	A	0.175		
rs4926499	1	2.49E+08	0.1803	G	C	0.295		
rs67720684	2	18975439	0.2084	A	A	0.204		
rs1344653	2	19730845	0.496	A	G	0.253		
rs7255	2	20878820	0.4714	T	T	0.192		
rs55701159	2	25139596	0.1402	G	T	0.396		
rs1275988	2	26914364	0.3706	C	C	0.541		
rs9678851	2	27887034	0.412	C	C	0.172		
rs7562	2	28635740	0.4471	C	T	0.231		
rs13408514	2	37203006	0.348	C	C	0.206		
rs13420463	2	37517566	0.2241	G	A	0.314		
rs7601637	2	37993304	0.2551	G	G	0.218		
rs2192768	2	40525932	0.4	T	T	0.231		
rs76326501	2	43167878	0.08792	C	A	0.527		
rs35590893	2	43716933	0.2691	A	G	0.215		
rs687914	2	45878760	0.2533	T	T	0.277		
rs6545155	2	50429861	0.1915	C	T	0.169		

rs10189186	2	53025757	0.4345	G	A	0.143		
rs2920899	2	55279681	0.2214	G	T	0.185		
rs13431149	2	56193665	0.408	A	A	0.206		
rs6730325	2	59315828	0.3844	G	G	0.11		
rs66723505	2	60063763	0.2734	G	A	0.213		
rs2540949	2	65284231	0.379	T	A	0.233	yes	rs111524356
rs2300481	2	66782467	0.3859	T	T	0.195		
rs6731373	2	68503044	0.3418	A	A	0.182		
rs72847885	2	86326717	0.3422	G	A	0.218		
rs57874285	2	1.12E+08	0.4145	A	A	0.156		
rs28377357	2	1.13E+08	0.3091	A	G	0.186		
rs11688682	2	1.21E+08	0.2882	C	G	0.222		
rs750416	2	1.22E+08	0.4175	T	C	0.15		
rs72844590	2	1.38E+08	0.1568	T	T	0.208		
rs10928240	2	1.46E+08	0.4012	G	G	0.216		
rs2848657	2	1.58E+08	0.1091	A	T	0.242		
rs79523138	2	1.61E+08	0.1084	G	G	0.263		
rs55732192	2	1.62E+08	0.1022	T	G	0.24		
rs73029563	2	1.65E+08	0.4281	C	C	0.358		
rs10184004	2	1.66E+08	0.435	T	C	0.205		
rs11694601	2	1.75E+08	0.4165	G	G	0.191		
rs55799551	2	1.77E+08	0.3765	G	G	0.203		
rs1837164	2	1.79E+08	0.3972	A	A	0.15		
rs12474446	2	1.83E+08	0.1824	A	T	0.276		
rs6739913	2	1.85E+08	0.2771	G	A	0.168		
rs28558491	2	1.88E+08	0.2455	C	C	0.187		
rs6434404	2	1.91E+08	0.3196	A	A	0.311		
rs61345672	2	1.92E+08	0.2382	C	A	0.228	yes	rs142457531

rs296797	2	2.01E+08	0.4271	T	T	0.165		
rs55780018	2	2.09E+08	0.4527	C	C	0.278		
rs1047891	2	2.12E+08	0.3122	A	C	0.196		
rs12694277	2	2.13E+08	0.2683	T	C	0.177		
rs1250259	2	2.16E+08	0.2567	T	T	0.452		
rs2571445	2	2.19E+08	0.3911	A	A	0.205		
rs1870123	2	2.19E+08	0.3713	G	G	0.153		
rs2943646	2	2.27E+08	0.3694	A	G	0.283		
rs10182307	2	2.28E+08	0.414	A	G	0.164		
rs1044822	2	2.31E+08	0.1263	T	C	0.228		
rs139354822	2	2.42E+08	0.0372	C	T	0.547		
rs347591	3	11290122	0.3424	G	T	0.318		
rs6793656	3	13823342	0.1433	G	G	0.266		
rs729639	3	13826854	0.3527	T	C	0.121		
rs11128722	3	14958126	0.4374	G	G	0.287		
rs189267552	3	20073193	0.01309	A	T	0.783		
rs4858758	3	20117233	0.4024	T	C	0.232		
rs2643826	3	27562988	0.4148	T	C	0.411		
rs12638085	3	30405936	0.3643	A	A	0.213		
rs267539	3	37582252	0.4791	A	G	0.178		
rs6788984	3	41107173	0.1309	G	A	0.256		
rs112912733	3	42054910	0.07859	A	C	0.421		
rs34877991	3	45643648	0.3992	C	G	0.173		
rs11716779	3	47606215	0.2379	G	A	0.271	yes	rs111499603
rs6774721	3	49381898	0.1402	A	G	0.252		
rs3821843	3	53558012	0.3192	G	G	0.334		
rs4681794	3	56776908	0.3112	C	C	0.211		
rs4499560	3	70920485	0.3197	A	T	0.201		

rs9857362	3	74710462	0.482	C	A	0.151		
rs1375564	3	85656311	0.338	C	T	0.234		
rs1882289	3	1.14E+08	0.122	G	G	0.216		
rs6438253	3	1.15E+08	0.4831	G	A	0.174		
rs3772840	3	1.25E+08	0.4203	T	C	0.185		
rs4854572	3	1.33E+08	0.4797	A	G	0.182		
rs863930	3	1.36E+08	0.4577	T	G	0.189		
rs357489	3	1.54E+08	0.2233	T	T	0.21		
rs78151625	3	1.58E+08	0.1587	C	C	0.211		
rs56394279	3	1.60E+08	0.4317	C	C	0.236		
rs12638862	3	1.69E+08	0.2725	G	A	0.258		
rs262986	3	1.83E+08	0.4587	A	G	0.186		
rs231708	4	2694773	0.2968	G	G	0.228		
rs2610990	4	18008232	0.2757	A	G	0.257		
rs4572866	4	26795603	0.3333	C	T	0.202		
rs2291435	4	38387395	0.493	C	C	0.262		
rs12511987	4	46595623	0.1805	G	G	0.215		
rs13141523	4	48789269	0.4458	A	G	0.195		
rs60991988	4	54801228	0.08603	G	T	0.318	yes	rs7671947
rs693367	4	56260662	0.319	G	G	0.178		
rs55940751	4	77365891	0.4516	T	C	0.212	yes	rs568240689
rs10857147	4	81181072	0.3454	T	A	0.63		
rs12649662	4	83896818	0.208	A	T	0.219		
rs2014912	4	86715670	0.1699	T	T	0.477		
rs13149209	4	89750668	0.2189	C	T	0.251		
rs1347345	4	95938386	0.3916	G	G	0.179		
rs17248480	4	1.02E+08	0.01795	A	G	0.733		
rs13107325	4	1.03E+08	0.04652	T	C	0.553		

rs144317085	4	1.06E+08	0.03712	T	T	0.451		
rs13112725	4	1.07E+08	0.2334	G	C	0.414		
rs78866112	4	1.09E+08	0.4087	A	A	0.188	yes	rs67372037
rs6815273	4	1.11E+08	0.4808	A	G	0.328		
rs3097937	4	1.25E+08	0.1593	T	A	0.159		
rs7439567	4	1.38E+08	0.4288	T	T	0.236		
rs72719160	4	1.44E+08	0.3211	T	T	0.234		
rs6823767	4	1.51E+08	0.2847	C	C	0.173		
rs3796592	4	1.57E+08	0.2207	C	T	0.295		
rs17035181	4	1.58E+08	0.1451	G	T	0.295		
rs869398	4	1.70E+08	0.4548	A	A	0.208		
rs4957026	5	361148	0.3198	A	A	0.198		
rs10069690	5	1279790	0.254	T	T	0.274		
rs1173771	5	32815028	0.4064	A	G	0.632		
rs74774746	5	33411769	0.2658	C	G	0.171		
rs1694068	5	53283630	0.387	T	A	0.234		
rs256904	5	55810305	0.2679	A	T	0.206		
rs13179413	5	55868097	0.2755	T	T	0.205		
rs10062049	5	61553881	0.1604	T	T	0.235		
rs6875372	5	64079015	0.4476	T	A	0.134		
rs3121685	5	65662133	0.4795	T	C	0.148		
rs1159201	5	66311339	0.2655	G	A	0.209		
rs246973	5	68007803	0.2954	T	T	0.224		
rs34797797	5	77853085	0.4577	A	G	0.247		
rs10059921	5	87514515	0.06495	T	G	0.425		
rs709668	5	96174186	0.2007	A	G	0.253		
rs1871190	5	97953719	0.3595	T	T	0.14		
rs10477931	5	1.08E+08	0.2294	C	A	0.212		

rs10077885	5	1.14E+08	0.4878	C	C	0.259		
rs4235771	5	1.14E+08	0.4878	A	G	0.241	yes	rs147202915
rs13359291	5	1.22E+08	0.1771	A	A	0.433		
rs62373688	5	1.27E+08	0.1165	A	A	0.261		
rs6595838	5	1.28E+08	0.3046	A	A	0.323		
rs702395	5	1.40E+08	0.4726	T	T	0.191		
rs10477176	5	1.42E+08	0.2606	G	A	0.185		
rs34070447	5	1.48E+08	0.4302	G	G	0.197		
rs1036190	5	1.48E+08	0.3962	G	G	0.217		
rs11953630	5	1.58E+08	0.3455	T	C	0.453		
rs1422799	5	1.58E+08	0.3653	G	G	0.263	yes	rs368910700
rs12153395	5	1.79E+08	0.1158	A	G	0.111		
rs2745599	6	1613686	0.441	G	A	0.191		
rs9349379	6	12903957	0.4291	G	A	0.225		
rs9368222	6	20686996	0.2702	A	A	0.23		
rs6911827	6	22130601	0.4735	T	T	0.238		
rs198851	6	26104632	0.1173	T	T	0.351		
rs6914824	6	27139048	0.1692	T	T	0.209		
rs76106973	6	27904345	0.02172	A	A	0.775		
rs7889	6	31605448	0.3785	C	G	0.25		
rs73744859	6	34208190	0.04579	C	C	0.375		
rs7763558	6	43349215	0.3144	A	A	0.353		
rs78648104	6	50683009	0.07857	C	C	0.429		
rs4712120	6	56092916	0.4375	G	T	0.197		
rs6454092	6	79701972	0.4903	A	G	0.228		
rs9449350	6	82281417	0.344	C	C	0.219		
rs35410524	6	96885405	0.1836	T	T	0.337		
rs2012071	6	1.12E+08	0.4426	A	G	0.158		

rs9401025	6	1.18E+08	0.4799	C	C	0.203		
rs1626650	6	1.19E+08	0.1146	T	C	0.307	yes	rs149379258
rs9401090	6	1.19E+08	0.25	C	T	0.164		
rs10782230	6	1.26E+08	0.4855	G	A	0.2		
rs9398815	6	1.27E+08	0.4594	C	T	0.361		
rs9885632	6	1.31E+08	0.2429	C	T	0.21		
rs12194247	6	1.34E+08	0.04857	T	C	0.378		
rs7763294	6	1.40E+08	0.3245	T	G	0.18		
rs7765526	6	1.48E+08	0.4784	A	A	0.201		
rs113397083	6	1.51E+08	0.06203	A	G	0.518		
rs2207232	6	1.52E+08	0.1295	C	C	0.375	yes	rs34862067
rs9356632	6	1.71E+08	0.1498	A	G	0.274		
rs6963853	7	1858725	0.4466	A	A	0.197		
rs2107595	7	19049388	0.1689	A	A	0.417		
rs12979	7	24738164	0.128	G	C	0.274		
rs6969780	7	27159136	0.095	C	C	0.296		
rs3735533	7	27245893	0.08335	T	C	0.652	yes	rs60772526
rs10274928	7	28142088	0.4926	A	A	0.159		
rs10233127	7	30933453	0.1054	A	A	0.303		
rs76206723	7	40447971	0.1241	A	G	0.412		
rs2075066	7	44265179	0.1523	A	A	0.251		
rs10260816	7	46010100	0.3972	G	G	0.351		
rs6593297	7	56122058	0.2731	A	A	0.158		
rs6963105	7	75097488	0.4332	A	G	0.178		
rs7796089	7	77512098	0.3201	C	G	0.221		
rs42377	7	92243672	0.3115	A	G	0.258		
rs17477177	7	1.06E+08	0.208	C	C	0.735		
rs4728142	7	1.29E+08	0.4479	A	G	0.181		

rs34072724	7	1.30E+08	0.4918	A	G	0.218		
rs13238550	7	1.31E+08	0.4038	A	A	0.257		
rs1860509	7	1.39E+08	0.1796	G	T	0.268		
rs12703989	7	1.40E+08	0.4729	A	A	0.152		
rs11771693	7	1.50E+08	0.3218	G	A	0.167		
rs3918226	7	1.51E+08	0.07065	T	T	0.509		
rs10224002	7	1.51E+08	0.274	G	G	0.367		
rs1870735	7	1.56E+08	0.492	C	C	0.17		
rs4875958	8	1721090	0.2751	G	A	0.226		
rs62491354	8	9730663	0.1413	A	A	0.257		
rs2898290	8	11433909	0.4717	T	T	0.312		
rs6557876	8	25900675	0.2623	T	C	0.416		
rs2979470	8	30288272	0.4705	T	T	0.162		
rs66963240	8	32392283	0.4302	T	T	0.19	yes	rs527953512
rs1906672	8	38130025	0.2117	A	A	0.272		
rs34473019	8	51704077	0.1942	T	T	0.252	yes	rs12547335
rs6996733	8	60535824	0.1554	C	T	0.188		
rs2354862	8	64501744	0.3512	C	A	0.192		
rs13253358	8	68920135	0.2833	T	T	0.173		
rs7837090	8	74221406	0.1816	G	C	0.22		
rs1449544	8	76591880	0.4354	C	A	0.219		
rs72688070	8	81393697	0.1453	T	C	0.241		
rs62526122	8	92769569	0.3178	A	A	0.211		
rs142449193	8	1.03E+08	0.03654	T	C	0.388		
rs35783704	8	1.06E+08	0.1264	A	G	0.462		
rs11775209	8	1.20E+08	0.2179	A	A	0.262	yes	rs566791419
rs56123029	8	1.25E+08	0.1823	C	C	0.192		
rs62523863	8	1.27E+08	0.2213	A	A	0.232		

rs4598218	8	1.29E+08	0.3799	C	T	0.18		
rs1036821	8	1.36E+08	0.2897	A	A	0.214		
rs4129585	8	1.43E+08	0.4362	A	A	0.175		
rs3802228	8	1.44E+08	0.4617	A	A	0.214		
rs520015	9	211762	0.498	G	C	0.12		
rs60191654	9	753648	0.1837	G	G	0.173		
rs28558845	9	4334791	0.1228	C	G	0.218		
rs1332813	9	9350706	0.3528	T	T	0.197		
rs111791351	9	19111187	0.1729	T	T	0.205		
rs2780841	9	20612516	0.2949	T	C	0.184		
rs9886665	9	22942770	0.259	T	T	0.151		
rs7042283	9	71518027	0.1442	G	G	0.22		
rs7045409	9	95201540	0.3612	A	T	0.182		
rs111245230	9	1.13E+08	0.03522	C	C	0.749		
rs10448275	9	1.17E+08	0.453	T	T	0.171		
rs16927927	9	1.28E+08	0.1363	C	C	0.282		
rs7023828	9	1.28E+08	0.392	T	C	0.244		
rs1891730	9	1.30E+08	0.3747	C	C	0.179		
rs184457	9	1.32E+08	0.295	A	G	0.166		
rs6271	9	1.37E+08	0.04954	T	C	0.411		
rs821317	9	1.40E+08	0.1571	A	A	0.2		
rs56352451	10	5804865	0.1434	T	T	0.199		
rs11256837	10	10840535	0.2083	A	A	0.211		
rs12258967	10	18727959	0.265	G	G	0.453		
rs3802517	10	28233469	0.4623	A	A	0.19		
rs34130368	10	48411796	0.03488	T	G	0.272		
rs72831344	10	63518378	0.1363	T	C	0.337		
rs7090758	10	65335315	0.4593	C	C	0.149		

rs11000752	10	75470355	0.1481	G	G	0.358		
rs4746172	10	75855842	0.284	C	C	0.171		
rs7096715	10	82195949	0.4316	T	T	0.172		
rs10788569	10	89604732	0.2473	C	T	0.228		
rs11187142	10	94468685	0.09415	T	T	0.331		
rs932764	10	95895940	0.4483	G	G	0.43		
rs57866767	10	96023077	0.3986	C	C	0.306		
rs4917612	10	96591284	0.1293	G	C	0.341	yes	rs199562446
rs112184198	10	1.03E+08	0.09226	A	G	0.663		
rs3218248	10	1.04E+08	0.01507	A	A	0.575		
rs11191548	10	1.05E+08	0.1065	C	T	1.098		
rs111790405	10	1.06E+08	0.01567	T	T	0.87		
rs7903146	10	1.15E+08	0.2668	T	T	0.221		
rs1801253	10	1.16E+08	0.2667	G	G	0.272		
rs11197813	10	1.19E+08	0.2973	G	G	0.18		
rs11592107	10	1.23E+08	0.2886	A	A	0.302		
rs72834453	10	1.24E+08	0.1261	G	G	0.276		
rs7912283	10	1.34E+08	0.3572	G	A	0.194		
rs1133400	10	1.34E+08	0.207	G	G	0.261		
rs12770172	10	1.34E+08	0.2026	G	A	0.252		
rs661348	11	1905292	0.444	C	C	0.445		
rs10743086	11	8774923	0.2023	A	G	0.183		
rs173396	11	9759918	0.3939	G	A	0.264	yes	rs58068637
rs7129220	11	10350538	0.104	A	A	0.5		
rs1401454	11	16250183	0.4099	T	T	0.55		
rs414992	11	16894090	0.1289	T	T	0.313		
rs757081	11	17351683	0.3483	G	G	0.343		
rs10766533	11	19224677	0.286	T	A	0.167		

rs871004	11	28512458	0.3719	A	A	0.2		
rs11031051	11	30355707	0.3132	C	C	0.19		
rs190194639	11	34068037	0.09707	T	T	0.275		
rs3740781	11	45255926	0.2687	C	T	0.226		
rs1585453	11	46884713	0.08309	T	T	0.389		
rs11039216	11	47406592	0.4851	C	T	0.313		
rs59652089	11	47907350	0.1519	T	C	0.404		
rs11040595	11	49878757	0.1363	T	G	0.278		
rs4385883	11	51539339	0.1848	T	A	0.233		
rs75905900	11	55113534	0.1333	C	A	0.388		
rs11229457	11	58207203	0.2374	T	C	0.317		
rs7125196	11	61272565	0.1368	C	C	0.264		
rs4980515	11	63744609	0.4961	C	T	0.209		
rs3741378	11	65408937	0.1262	T	C	0.409		
rs67976715	11	68023742	0.2035	C	C	0.197		
rs11263441	11	69252000	0.07423	A	G	0.402		
rs72931748	11	69825414	0.09354	G	A	0.3		
rs7927515	11	76125330	0.3425	A	A	0.227		
rs10830278	11	89216425	0.2078	T	A	0.259		
rs10895001	11	1.01E+08	0.2938	A	G	0.374	yes	rs35571646
rs4754196	11	1.07E+08	0.4997	G	G	0.288		
rs7128382	11	1.17E+08	0.1782	A	A	0.269		
rs896693	11	1.20E+08	0.4133	A	G	0.166		
rs1106243	11	1.23E+08	0.4864	G	G	0.181		
rs11222084	11	1.30E+08	0.3452	T	T	0.299		
rs11571376	12	1059556	0.3075	G	G	0.188		
rs736107	12	12627410	0.358	A	G	0.173		
rs2024385	12	12888438	0.3885	A	T	0.233		

rs28621435	12	13860990	0.1032	A	G	0.193		
rs60691990	12	20368269	0.3116	C	T	0.353		
rs7976167	12	24210599	0.2997	C	T	0.164		
rs11168516	12	48702065	0.1733	T	T	0.247		
rs1126930	12	49399132	0.03367	C	C	0.638		
rs10747570	12	50509937	0.3672	A	A	0.293		
rs61926181	12	50767037	0.04959	A	G	0.547		
rs73099903	12	53440779	0.08538	T	T	0.469		
rs7297416	12	54443090	0.3362	C	A	0.376		
rs10437954	12	58003922	0.09034	G	G	0.347		
rs9669278	12	66374587	0.4838	C	T	0.217	yes	rs113744258
rs4143175	12	67782397	0.2438	T	T	0.18		
rs7963801	12	79685226	0.4358	T	C	0.203		
rs7964067	12	79904658	0.1663	A	A	0.25		
rs17249754	12	90060586	0.1451	A	G	0.845		
rs10858966	12	90567026	0.3234	C	C	0.254		
rs5742643	12	1.03E+08	0.2464	T	C	0.224		
rs11064687	12	1.10E+08	0.03826	G	G	0.378		
rs11066320	12	1.13E+08	0.4373	A	A	0.246		
rs2384550	12	1.15E+08	0.3534	A	G	0.168		
rs35444	12	1.16E+08	0.4061	G	G	0.367		
rs117206641	12	1.33E+08	0.1199	T	T	0.216		
rs2480171	13	21559858	0.1358	T	T	0.205		
rs606950	13	22298923	0.3573	G	A	0.232		
rs1331012	13	27115424	0.2622	T	T	0.204		
rs9508495	13	30146201	0.2597	C	C	0.224		
rs9603376	13	32181957	0.4807	C	A	0.326		
rs4274337	13	41967193	0.1612	A	G	0.22		

rs9532959	13	42543516	0.07405	A	A	0.314		
rs73187288	13	42738672	0.1052	C	C	0.256		
rs912434	13	47189928	0.2581	G	T	0.215		
rs9526707	13	51489186	0.3094	A	G	0.194		
rs75961402	13	56398286	0.1666	A	A	0.182		
rs78474310	13	73826901	0.04428	G	G	0.483		
rs7988232	13	79808655	0.4407	A	A	0.148		
rs9549328	13	1.14E+08	0.2281	T	T	0.267		
rs7331680	13	1.15E+08	0.1461	T	T	0.386		
rs17115145	14	30122409	0.3934	T	T	0.157		
rs8904	14	35871217	0.3895	A	A	0.212		
rs34983854	14	39858442	0.3973	G	G	0.162		
rs72683923	14	50735947	0.01722	C	T	0.977		
rs9888615	14	53377540	0.2937	T	C	0.274		
rs8016306	14	63928546	0.2088	G	A	0.189		
rs11623535	14	72462381	0.2654	G	A	0.202		
rs11622562	14	77517148	0.3163	T	C	0.176		
rs8009025	14	98631171	0.1903	C	T	0.283		
rs10149871	14	1.00E+08	0.4545	T	T	0.213		
rs8014182	14	1.04E+08	0.1473	T	C	0.34		
rs11629850	15	40317075	0.4643	G	A	0.2		
rs2925345	15	41311799	0.4309	T	T	0.234		
rs1018148	15	48903126	0.08125	A	G	0.431		
rs1440371	15	66941084	0.2887	A	A	0.217		
rs72756346	15	69597932	0.2723	T	G	0.205		
rs1378942	15	75077367	0.3229	C	C	0.324		
rs11634028	15	76276150	0.1782	A	A	0.226		
rs2759308	15	81016227	0.4736	A	A	0.316		

rs3743157	15	85680532	0.1596	A	A	0.26		
rs11632436	15	86295286	0.4781	G	C	0.206		
rs2071382	15	91428197	0.4632	T	T	0.409		
rs62020769	15	93280491	0.4725	C	C	0.174		
rs12906962	15	95312071	0.3109	C	T	0.232		
rs4965529	15	1.00E+08	5.12E-05	T	C	0.25		
rs11248866	16	1365341	0.359	G	G	0.173		
rs140869992	16	2158570	0.007765	A	G	1.472		
rs2379829	16	3538873	0.2779	G	G	0.202		
rs1034906	16	4128853	0.1551	C	G	0.207		
rs77997837	16	4944256	0.2158	T	T	0.267	yes	rs558074929
rs35450617	16	6889675	0.3322	G	G	0.161		
rs2188717	16	24730230	0.1988	T	C	0.332		
rs34294937	16	49883927	0.228	A	A	0.208		
rs34941092	16	50550137	0.156	A	G	0.23		
rs62049427	16	70334172	0.0571	A	A	0.559		
rs1012089	16	74171973	0.4618	C	G	0.131		
rs12449170	16	75463012	0.4107	C	T	0.302		
rs7187540	16	85318302	0.36	A	C	0.177		
rs731749	16	86356582	0.08937	A	A	0.32		
rs3851018	16	86437811	0.4256	G	G	0.118		
rs6540125	16	87993889	0.3485	T	T	0.164		
rs10852858	17	1357751	0.4551	T	T	0.229		
rs4790309	17	1966889	0.4264	T	C	0.244	yes	rs12952051
rs12937989	17	6479453	0.4012	A	G	0.231		
rs2428362	17	7180274	0.4267	C	T	0.241		
rs62059712	17	7740170	0.0776	C	T	0.394		
rs4925159	17	18185510	0.4246	A	A	0.228		

rs1551355	17	30032420	0.2547	T	T	0.193		
rs9899540	17	30777924	0.414	A	A	0.201		
rs12946454	17	43208121	0.2465	T	T	0.413		
rs17608766	17	45013271	0.1429	C	C	0.497		
rs7406910	17	46688256	0.09442	T	C	0.435		
rs9912738	17	47510584	0.3275	C	T	0.204		
rs34430710	17	56876627	0.2832	T	T	0.207		
rs1292058	17	57957982	0.2532	A	T	0.277		
rs1036902	17	58950791	0.1611	C	C	0.227		
rs8068318	17	59483766	0.2603	C	T	0.42		
rs8078112	17	60720362	0.3891	C	T	0.191		
rs4291	17	61554194	0.3823	T	T	0.206		
rs112260610	17	64252393	0.1366	T	T	0.222		
rs3744010	17	73840243	0.2211	A	G	0.276		
rs9302885	17	76799898	0.4435	A	A	0.195		
rs112280096	17	79367409	0.3694	A	C	0.171		
rs34413141	18	777282	0.1763	A	T	0.308		
rs1154214	18	24546824	0.4108	T	G	0.203		
rs356833	18	26323525	0.2281	G	A	0.172		
rs7226575	18	31168982	0.07087	C	C	0.3		
rs7245273	18	42074522	0.3334	A	G	0.235		
rs12958173	18	42141977	0.2916	A	A	0.295		
rs1025686	18	48147127	0.2547	G	A	0.214		
rs11876341	18	48799991	0.2853	G	G	0.223		
rs599550	18	53252388	0.1465	G	G	0.283		
rs10048404	18	54578482	0.3719	T	C	0.228		
rs6567160	18	57829135	0.2349	C	T	0.154		
rs12454712	18	60845884	0.4028	C	T	0.204		

rs10460108	18	73034151	0.4899	A	A	0.212		
rs3810409	19	2180515	0.05024	A	A	0.645	yes	rs576345045
rs2613765	19	5066330	0.4553	A	G	0.234		
rs4247374	19	7252756	0.1404	T	C	0.635		
rs10409243	19	10332988	0.44	C	C	0.113		
rs167479	19	11526765	0.4641	T	G	0.412		
rs17638167	19	11584818	0.04712	T	C	0.478		
rs8104223	19	15363477	0.2937	G	A	0.214		
rs3745348	19	17212410	0.2706	T	T	0.26		
rs8104559	19	22205167	0.153	C	C	0.228		
rs8105753	19	31927547	0.3713	C	A	0.218		
rs7256564	19	33889593	0.3135	A	A	0.153		
rs7412	19	45412079	0.0785	T	C	0.326		
rs10423928	19	46182304	0.2252	A	A	0.198		
rs73046792	19	49605705	0.175	A	G	0.282		
rs138877676	19	50935809	0.01611	T	G	0.656		
rs1764975	20	4101290	0.2099	T	A	0.216		
rs6054200	20	6365645	0.4211	G	A	0.164		
rs1887320	20	10965998	0.4536	A	A	0.311		
rs6141767	20	31225069	0.1751	C	C	0.286		
rs6031435	20	42797358	0.459	G	G	0.259		
rs6021247	20	50108980	0.4967	A	A	0.204		
rs6026739	20	57739469	0.1342	T	T	0.39		
rs13039439	20	62703490	0.2708	T	C	0.326	yes	rs146216988
rs1882961	21	16556367	0.3305	T	T	0.235		
rs11701033	21	33788341	0.1903	G	G	0.219		
rs220249	21	43472148	0.4319	A	A	0.159		
rs162366	21	44935383	0.4039	A	G	0.242		

rs758374	22	19971552	0.3302	C	C	0.21		
rs9608690	22	28921347	0.06859	A	G	0.371		
rs12321	22	29453193	0.4149	C	G	0.195		
rs28578714	22	50727921	0.4193	C	T	0.195		

*the reported minor allele frequency (MAF) is computed from MDC; §Beta refers to the one reported in the reference GWAS study

S2 List of SNPs with weights and effect allele used to compute the DBP-GRS_{384}

**rsID**	**Chr**	**BP**	**MAF***	**Minor Allele**	**Effect allele**	**Beta§**	**proxy**	**lead snps**
rs4970449	1	1353632	0.075433	G	A	0.4383		
rs2493292	1	3328659	0.141947	T	T	0.2481		
rs4908678	1	7739250	0.393976	C	C	0.1124		
rs191602286	1	11843008	4.05E-05	A	C	0.3061		
rs6686889	1	25030470	0.246695	T	T	0.1918		
rs1565716	1	29549216	0.06238	A	A	0.2012		
rs4660293	1	40028180	0.251672	G	G	0.0998		
rs12142296	1	46541679	0.129386	G	G	0.1716		
rs4926923	1	48109225	0.076699	C	T	0.1918		
rs6681713	1	51527684	0.032502	G	T	0.4074		
rs34370185	1	59656909	0.307772	T	T	0.2019		
rs34517439	1	78450517	0.116021	A	C	0.233		
rs2065152	1	90228519	0.363376	T	T	0.1103		
rs2932538	1	113216543	0.261439	A	G	0.237		
rs12078697	1	117015118	0.199797	C	G	0.1083		
rs1886914	1	119541452	0.402619	C	G	0.1106		
rs72704264	1	145713305	0.20785	C	C	0.1173		
rs13796	1	154245917	0.128077	C	C	0.1626		
rs2171690	1	164740099	0.481106	C	T	0.1181		

rs7524019	1	167367193	0.495012	C	T	0.1036		
rs12405515	1	172357441	0.446425	G	G	0.1698		
rs150816167	1	179571862	0.040363	C	C	0.2873		
rs41475048	1	183058452	0.243886	G	G	0.1233		
rs882624	1	201735913	0.333046	T	C	0.1536		
rs2169137	1	204497913	0.247024	G	C	0.1588		
rs2629665	1	207220800	0.421806	A	C	0.1193		
rs12408022	1	217718789	0.275901	T	T	0.1483		
rs35981664	1	218549354	0.306069	T	T	0.1542		
rs9431431	1	221358796	0.31067	A	G	0.134		
rs2760061	1	228191075	0.492233	A	A	0.1771		
rs2004776	1	230848702	0.236073	T	T	0.2513		
rs6429422	1	243472801	0.308838	G	G	0.246		
rs56236159	2	3636478	0.151802	G	T	0.1265		
rs10176996	2	19708692	0.29895	C	C	0.2275		
rs13035244	2	25134009	0.41315	C	T	0.1606		
rs1607644	2	34679626	0.374649	A	G	0.1035		
rs2707238	2	38094149	0.279291	C	C	0.1019		
rs4952611	2	40567743	0.394708	C	C	0.1401		
rs11681462	2	42352567	0.199653	C	C	0.1325		
rs76326501	2	43167878	0.088217	C	A	0.3618		
rs17737768	2	46357133	0.111623	C	C	0.2621		
rs1975487	2	55809054	0.465585	A	G	0.141		
rs11899888	2	56102744	0.145757	G	G	0.2549		
rs7608483	2	61836235	0.409345	A	A	0.1171		
rs13014371	2	64217786	0.428449	C	C	0.1176		
rs12052761	2	69065841	0.398142	A	G	0.1229		
rs10193543	2	72483329	0.151307	G	T	0.1391		
rs1876487	2	73114352	0.314925	A	C	0.1099		

rs2579519	2	96675166	0.399223	C	C	0.1818		
rs4851462	2	98357163	0.42154	C	C	0.1138		
rs6722745	2	108875244	0.306198	C	C	0.0926		
rs62158170	2	114082175	0.212676	G	A	0.1645		
rs10864859	2	121440218	0.071011	G	T	0.1962		
rs13001283	2	127183454	0.171093	A	A	0.1513		
rs4954192	2	135632981	0.367253	T	C	0.1225		
rs7606205	2	144146311	0.292111	C	C	0.1207		
rs1438896	2	145646072	0.299096	T	T	0.195		
rs34570306	2	146272860	0.467443	C	C	0.1115		
rs62169544	2	146950908	0.437223	A	G	0.1207		
rs12990959	2	148572160	0.327074	C	C	0.1271		
rs1220128	2	158499902	0.127444	G	C	0.1733		
rs2390258	2	166250129	0.271766	A	G	0.1031		
rs6758859	2	173965056	0.34808	C	T	0.1211		
rs79146658	2	179786068	0.081127	C	C	0.3344		
rs10184839	2	181946115	0.258804	A	T	0.1398		
rs16823124	2	183224127	0.321632	A	A	0.2276		
rs7592578	2	191439591	0.193426	T	G	0.1998		
rs2162003	2	205077128	0.401268	C	T	0.1279		
rs1263671	2	207996447	0.166263	C	C	0.1394		
rs1250259	2	216300482	0.256604	T	T	0.4275		
rs4674114	2	217659266	0.191005	A	G	0.1808		
rs1063281	2	218668732	0.397139	C	C	0.1623		
rs1996992	2	219651349	0.049884	T	G	0.2964		
rs12474050	2	220362557	0.341635	T	T	0.1142		
rs4507125	2	239864732	0.216987	C	C	0.1244		
rs9865843	3	7489993	0.47353	G	G	0.0906		
rs4634143	3	23163749	0.303272	T	T	0.1129		

rs13082711	3	27537909	0.218582	C	C	0.1778		
rs72851229	3	29374219	0.166267	C	G	0.1364		
rs3774372	3	41877414	0.13025	C	C	0.0254		
rs2683696	3	41914898	0.15742	C	C	0.3962		
rs113134141	3	46861939	0.103032	G	G	0.1569		
rs13094615	3	47534921	0.001151	T	T	0.4073		
rs6797587	3	48197614	0.294463	A	G	0.2378		
rs36022378	3	49913705	0.191956	C	C	0.1765		
rs9821489	3	53575728	0.006385	A	A	0.4962		
rs9810888	3	53635595	0.49526	G	G	0.1151		
rs9827472	3	56726646	0.361308	T	C	0.133		
rs12486605	3	57706503	0.448835	C	C	0.1437		
rs3774702	3	63856870	0.172738	A	A	0.147		
rs918466	3	64710253	0.389234	A	G	0.1402		
rs11923667	3	101268080	0.435855	A	A	0.1222		
rs28675079	3	111500002	0.209867	A	G	0.1444		
rs55914222	3	128202943	0.028185	C	G	0.5104		
rs55688777	3	133886705	0.345455	C	T	0.18		
rs2306374	3	138119952	0.157434	C	C	0.1774		
rs16851397	3	141134818	0.047537	G	G	0.3942		
rs73158427	3	153721493	0.143453	A	A	0.1801		
rs419076	3	169100886	0.461669	T	T	0.2755		
rs7611674	3	179169230	0.179973	G	T	0.1576		
rs12374077	3	185317674	0.333901	C	C	0.1748		
rs1706003	3	194299967	0.451296	T	T	0.1326		
rs6777317	3	197070959	0.303371	A	A	0.1147		
rs55829085	4	2165493	0.046635	C	C	0.2672		
rs28667801	4	26785356	0.423067	T	T	0.1449		
rs1878825	4	36091370	0.374266	G	G	0.1072		

rs1718845	4	57943153	0.28793	A	G	0.0963		
rs6551716	4	63575696	0.121686	T	A	0.1478		
rs10008637	4	77414144	0.47397	C	T	0.1879		
rs16998073	4	81184341	0.346192	T	T	0.494		
rs7694000	4	95324968	0.464727	T	T	0.0954		
rs17248480	4	102435265	0.01819	A	G	0.5064		
rs13107325	4	103188709	0.046802	T	C	0.6747		
rs223361	4	103769304	0.3446	C	T	0.1522		
rs7694643	4	109017528	0.338491	G	G	0.1209		
rs6825911	4	111381638	0.183784	C	C	0.202		
rs4834735	4	119958809	0.148133	T	T	0.1511		
rs66887589	4	120509279	0.467967	C	C	0.161		
rs4292285	4	145271954	0.40244	A	T	0.1073		
rs13139571	4	156645513	0.221889	A	C	0.2408		
rs954767	5	3706050	0.261132	C	C	0.1389		
rs12515541	5	57095011	0.360721	G	T	0.1104		
rs1848510	5	57754005	0.369312	A	A	0.1256		
rs10062049	5	61553881	0.160459	T	T	0.2113		
rs4286632	5	66291370	0.26862	G	A	0.127		
rs10078021	5	75038431	0.381734	G	G	0.1534		
rs62380354	5	89484911	0.111144	C	A	0.1825		
rs1579036	5	122921103	0.330477	A	A	0.1714		
rs6891344	5	123136656	0.173543	G	A	0.2182		
rs12521868	5	131784393	0.420122	T	G	0.1403		
rs55747751	5	132397351	0.102144	A	G	0.2159		
rs34070447	5	147871508	0.430073	G	G	0.1126		
rs9687065	5	148391140	0.193716	G	A	0.2199		
rs114503346	5	172192350	0.049831	T	C	0.2678		
rs72812846	5	173377636	0.308508	A	T	0.2053		

rs28362590	5	176731452	0.242509	G	T	0.1242		
rs1799945	6	26091179	0.117273	G	G	0.3888		
rs13191474	6	27413345	0.097389	T	C	0.326	yes	rs547190239
rs926552	6	29548089	0.116591	A	G	0.2501		
rs805303	6	31616366	0.37247	A	G	0.228		
rs115245297	6	34244132	0.044035	C	C	0.3148		
rs4714224	6	39186743	0.280026	C	G	0.139		
rs9472135	6	43809802	0.285176	C	T	0.1444		
rs13205180	6	51832494	0.4978	C	T	0.1721		
rs12203179	6	55994237	0.14248	G	C	0.219		
rs504691	6	72206620	0.402901	A	C	0.1177		
rs10943605	6	79655477	0.490872	A	A	0.1723		
rs7753695	6	80818531	0.439772	T	T	0.1031		
rs72613227	6	106320771	0.136566	T	T	0.1884		
rs3822857	6	116313931	0.316439	C	G	0.1194		
rs2693560	6	117523671	0.346764	A	G	0.1475		
rs9372498	6	118572486	0.082993	A	A	0.2731		
rs12208772	6	122125427	0.1107	C	C	0.2679		
rs13209747	6	127115454	0.435207	T	T	0.3017		
rs668459	6	139835689	0.441517	C	C	0.1219		
rs17080102	6	151004770	0.062101	C	G	0.4853		
rs7766549	6	152395963	0.133134	C	T	0.2259		
rs9479509	6	153427265	0.304973	A	G	0.1152		
rs598682	6	154418759	0.255937	A	G	0.1153		
rs9456648	6	161712235	0.371419	T	C	0.1166		
rs1322639	6	169587103	0.231289	G	A	0.2352		
rs73033340	7	1195692	0.048191	G	A	0.4615		
rs6959688	7	1966831	0.408648	G	G	0.2002		
rs2969070	7	2512545	0.352185	G	G	0.1791		

rs73049928	7	4669949	0.220412	G	G	0.1167		
rs1468520	7	7290732	0.16648	G	G	0.1638		
rs13240040	7	14375977	0.294079	G	A	0.1186		
rs12705091	7	19045397	0.217704	G	G	0.2078		
rs4507656	7	22156538	0.320498	G	G	0.1487		
rs342989	7	35467896	0.195604	A	A	0.1631		
rs73105827	7	45036785	0.085053	T	G	0.1878		
rs2854746	7	45960645	0.372084	C	C	0.228		
rs17454517	7	50915776	0.476898	A	A	0.1216		
rs2222544	7	69769369	0.259075	C	C	0.0987		
rs34324971	7	74107374	0.175109	A	A	0.1199		
rs2282978	7	92264410	0.340892	C	C	0.1578		
rs1947228	7	96461649	0.412947	T	C	0.1451		
rs2526620	7	100472380	0.203563	T	C	0.2072	yes	rs6963129
rs2392929	7	106414069	0.208103	G	G	0.2573		
rs11556924	7	129663496	0.364048	T	C	0.181		
rs1722886	7	134215259	0.432908	T	A	0.1166		
rs3918226	7	150690176	0.070551	T	T	0.6117		
rs891511	7	150704843	0.347295	A	G	0.2109		
rs9638084	7	156311745	0.406978	A	A	0.1107		
rs2922895	8	6379932	0.429711	C	C	0.1317		
rs75902664	8	17427186	0.01494	G	G	0.3546		
rs1047030	8	22428708	0.204129	G	A	0.1291		
rs62503324	8	23400615	0.208119	T	T	0.1931		
rs17321041	8	26445194	0.076001	T	T	0.2313		
rs56345595	8	82814156	0.428905	G	A	0.1887		
rs2142141	8	90940205	0.475115	G	G	0.1054		
rs2978098	8	101676675	0.44256	C	A	0.1548		
rs2513877	8	103883630	0.208372	A	G	0.1294		

rs11780200	8	120458521	0.218064	A	G	0.3319		
rs894344	8	135612745	0.406858	G	G	0.1267		
rs6578061	8	141045732	0.43071	G	A	0.1465		
rs10087782	8	141858620	0.465864	T	T	0.1211		
rs34591516	8	142367087	0.059714	T	T	0.3121		
rs62524579	8	144060955	0.465844	G	G	0.1656		
rs56233017	8	144981488	0.040108	A	G	0.2669		
rs12216886	9	2493751	0.189108	G	T	0.1292		
rs35287509	9	10594635	0.319876	C	C	0.1082		
rs4364717	9	21801530	0.459499	G	G	0.1006		
rs1537373	9	22103341	0.46054	G	G	0.1397	yes	rs9644861
rs76452347	9	35906471	0.184016	T	C	0.2246		
rs11141731	9	89888472	0.217398	T	C	0.1258		
rs7020564	9	109670016	0.306413	T	T	0.1105		
rs7043304	9	112358150	0.155317	C	T	0.176		
rs1861881	9	119312256	0.326402	T	T	0.115		
rs687621	9	136137065	0.377322	G	A	0.1327		
rs6271	9	136522274	0.056065	T	C	0.4313		
rs11145807	9	139520789	0.420268	A	A	0.155		
rs10906391	10	13523937	0.357318	T	T	0.121		
rs4373814	10	18419972	0.442149	C	C	0.1595		
rs1813353	10	18707448	0.305129	C	T	0.2772		
rs1265842	10	28924901	0.481835	T	T	0.1113		
rs2505084	10	30335520	0.468007	A	G	0.1735		
rs76164690	10	32590362	0.1337	G	G	0.154		
rs2246438	10	45273079	0.287325	A	G	0.1119		
rs10761530	10	62390726	0.46494	C	T	0.0955		
rs1530440	10	63524591	0.188203	T	C	0.4983		
rs10995311	10	64564934	0.450133	G	C	0.2017		

rs77413490	10	89681688	0.040155	T	T	0.473		
rs4494250	10	96563757	0.390894	A	A	0.1917		
rs603424	10	102075479	0.163816	A	A	0.1717		
rs72847884	10	103115345	0.048852	G	A	0.2664		
rs4387287	10	105677897	0.162375	A	A	0.1575		
rs191784289	10	106894942	0.016962	T	T	0.7062		
rs111777102	10	111965826	0.059325	T	T	0.214		
rs1801253	10	115805056	0.266754	G	C	0.3183		
rs72842207	10	121433675	0.227719	T	C	0.2112		
rs4411245	10	126712781	0.28015	A	A	0.1023		
rs17224476	11	4673788	0.084925	A	A	0.1547		
rs2929184	11	6289118	0.24145	G	A	0.1164		
rs110419	11	8252853	0.474901	G	A	0.112		
rs360153	11	9762274	0.392363	T	C	0.1917		
rs900145	11	13293905	0.297278	C	T	0.1493		
rs4757391	11	16302939	0.204652	C	C	0.3041		
rs381815	11	16902268	0.2818	T	T	0.2043		
rs11026586	11	22515533	0.08118	A	A	0.2606		
rs11030119	11	27728102	0.305642	A	G	0.1679		
rs919045	11	31111810	0.372589	C	T	0.1193		
rs7103648	11	47461783	0.368836	G	G	0.235		
rs751984	11	61278246	0.139313	C	T	0.3937		
rs4601790	11	65353906	0.280776	G	A	0.0197		
rs67330701	11	69079707	0.094989	T	C	0.2798		
rs875106	11	70005641	0.44002	G	G	0.1217		
rs504217	11	72006086	0.071082	T	T	0.2745		
rs2298807	11	73068571	0.227543	C	T	0.1233		
rs4420291	11	74374950	0.493583	G	A	0.0937		
rs59986178	11	77359909	7.94E-05	A	A	0.176		

rs2450128	11	77940075	0.161079	A	G	0.1505		
rs2289124	11	89224477	0.162229	A	A	0.2199		
rs11021221	11	95308854	0.170172	A	T	0.1772		
rs633185	11	100593538	0.299964	G	C	0.376		
rs61892344	11	101100768	0.141921	T	C	0.1162		
rs12362593	11	111586091	0.266686	G	G	0.1275		
rs12797557	11	117254122	0.284525	A	A	0.177		
rs12574332	11	122521123	0.126612	T	T	0.1747		
rs11222084	11	130273230	0.34529	T	A	0.2736		
rs78998485	12	434755	0.254179	G	G	0.2036		
rs55935819	12	2521579	0.345125	A	A	0.1088		
rs75507123	12	5417856	0.118319	T	G	0.1396		
rs7132012	12	8832203	0.318546	G	A	0.148		
rs7313556	12	15297359	0.330353	A	A	0.09		
rs61912333	12	19554817	0.467387	G	C	0.1191		
rs12579720	12	20173764	0.205628	C	G	0.2865		
rs6487543	12	26438189	0.227637	G	A	0.1325		
rs1098708	12	27321112	0.472014	G	G	0.0931		
rs7965392	12	42540280	0.386763	A	A	0.0972		
rs11168245	12	48204499	0.242441	G	C	0.1822		
rs7302981	12	50537815	0.367388	A	A	0.2652		
rs7137749	12	57098040	0.340742	T	T	0.132		
rs149723171	12	94830475	0.073968	T	T	0.2933	yes	rs144449115
rs11108209	12	96109855	0.082504	C	C	0.1901		
rs7134060	12	96717095	0.426163	A	G	0.0999		
rs12184466	12	111281636	0.203667	T	T	0.2533		
rs3184504	12	111884608	0.478431	T	T	0.4999		
rs10850411	12	115387796	0.29462	C	T	0.1856		
rs35444	12	115552437	0.406087	G	A	0.2671		

rs11067763	12	116198341	0.102446	G	A	0.2177		
rs3898618	12	120813921	0.050546	C	C	0.2532		
rs28498002	12	122599796	0.464366	T	C	0.1566		
rs1060105	12	123806219	0.19944	T	C	0.1894		
rs1271309	12	124820705	0.177109	A	G	0.1786		
rs55641580	13	25257917	0.124233	T	T	0.1745		
rs9549297	13	41397482	0.192124	G	G	0.1472		
rs12583637	13	50564490	0.0024	T	G	0.1671		
rs9563529	13	58316637	0.220739	T	T	0.1173		
rs3861113	13	72364382	0.082678	A	A	0.2126		
rs1215469	13	80707408	0.211138	A	C	0.1219		
rs55684003	13	97988689	0.282674	G	A	0.1193		
rs7987651	13	110448501	0.161614	T	C	0.1329		
rs17880989	14	23313633	0.039399	A	A	0.3648		
rs365990	14	23861811	0.334095	G	A	0.1937		
rs4424827	14	35110857	0.40467	C	C	0.0948		
rs11628933	14	60700903	0.230138	C	G	0.1338		
rs731681	14	68010224	0.433024	G	G	0.1071		
rs57786342	14	69260028	0.198211	A	A	0.1423		
rs8014161	14	92393198	0.37594	A	A	0.2163		
rs34161718	14	104620193	0.25427	T	C	0.1319		
rs10873612	15	26105602	0.399625	C	C	0.1096		
rs4924570	15	41974660	0.380448	C	C	0.1548		
rs9806323	15	48766015	0.145861	A	A	0.2172	yes	rs60317590
rs956006	15	62808539	0.328937	T	T	0.1595		
rs7178615	15	66869072	0.383166	A	G	0.1371		
rs62004794	15	68454523	0.463028	A	G	0.0948		
rs11853359	15	71621524	0.311254	A	G	0.1608		
rs61653296	15	74557817	0.189348	G	G	0.1405		

rs1378942	15	75077367	0.322906	C	C	0.388		
rs62012628	15	79070000	0.316023	T	C	0.238		
rs2034618	15	83799632	0.247496	T	C	0.1157		
rs7180952	15	85162551	0.439804	C	C	0.0984		
rs2521501	15	91437388	0.325307	T	T	0.3693		
rs873122	15	92702020	0.25826	G	C	0.1147		
rs12906962	15	95312071	0.310508	C	C	0.2378		
rs4984496	15	96635898	0.308955	T	T	0.1584		
rs9932866	16	706067	0.411735	A	A	0.1098		
rs28590346	16	2080653	0.337803	T	T	0.1786		
rs12921187	16	4943019	0.4099	T	G	0.175		
rs57327054	16	14487036	0.297027	T	C	0.1173		
rs59333122	16	19149996	0.276703	A	A	0.1087		
rs13333226	16	20365654	0.180911	G	A	0.2965		
rs6565174	16	30111904	0.101931	A	C	0.1821		
rs72799341	16	30936743	0.23639	A	A	0.1599		
rs10468291	16	49768046	0.432706	C	C	0.1166		
rs9932220	16	51758116	0.227639	A	G	0.1591		
rs12598649	16	56320824	0.336249	A	G	0.1519	yes	rs62039274
rs45474499	16	66914492	0.057502	T	T	0.3433		
rs7185555	16	69131281	0.137359	C	G	0.1537		
rs77870048	16	69965021	0.053516	T	C	0.4982		
rs8059962	16	81574197	0.421412	T	C	0.1397		
rs7500448	16	83045790	0.241346	G	A	0.2134		
rs1126464	16	89704365	0.266397	C	C	0.2071		
rs7215084	17	3880148	0.481251	C	T	0.1196		
rs2009598	17	6470598	0.422221	A	G	0.1674		
rs78378222	17	7571752	0.01741	G	G	1.0386		
rs8069739	17	8078765	0.300509	T	C	0.1082		

rs941454	17	18230380	0.441475	G	C	0.1569		
rs12938803	17	19204432	0.177746	A	C	0.1598		
rs11080134	17	29161503	0.346931	G	G	0.0974		
rs11874	17	45017193	0.129433	A	G	0.2327	yes	rs533030436
rs16948048	17	47440466	0.381388	G	A	0.26		
rs3786130	17	60765043	0.423476	C	T	0.1571		
rs4308	17	61559625	0.380651	A	A	0.1753		
rs6504213	17	62381714	0.43557	T	C	0.2441		
rs2467099	17	73949045	0.206695	T	C	0.1428		
rs57927100	17	75317300	0.253476	G	C	0.206		
rs11665020	18	10879503	0.293681	C	G	0.1371		
rs4800420	18	20158965	0.297193	A	A	0.1114		
rs10164193	18	31161426	0.069097	G	G	0.2196		
rs2543029	18	43098275	0.1788	C	A	0.2381		
rs745821	18	48142854	0.259125	G	T	0.189		
rs34163044	18	51851616	0.431217	A	A	0.1488		
rs72930904	18	52607301	0.15848	T	C	0.14		
rs12605156	18	53498114	0.206913	T	A	0.1418		
rs7235890	18	55732115	0.108485	G	G	0.1692		
rs2009733	19	8398714	0.499828	G	A	0.1054		
rs167479	19	11526765	0.465953	T	G	0.362		
rs3745318	19	16436262	0.241483	T	T	0.1396		
rs2304130	19	19789528	0.111468	G	G	0.2396		
rs6511291	19	21950402	0.462062	T	C	0.1158		
rs62104477	19	30294991	0.330012	T	T	0.1703		
rs1821295	19	32590773	0.322702	C	C	0.1347		
rs12983238	19	39438532	0.293996	A	G	0.1266		
rs6108168	20	8626271	0.250207	A	C	0.1901		
rs1327235	20	10969030	0.453974	G	G	0.3018		

rs1232482	20	11886643	0.388094	T	C	0.1155		
rs2618647	20	17882452	0.465066	A	G	0.0983		
rs3790228	20	19469150	0.271277	G	G	0.1759		
rs6060114	20	30169673	0.161298	C	T	0.1689		
rs13042148	20	32298286	0.139193	T	C	0.1543		
rs4810332	20	40268334	0.376395	A	T	0.1562		
rs6095241	20	47308798	0.413868	A	G	0.1358		
rs237485	20	48004238	0.293355	G	A	0.1124		
rs6015450	20	57751117	0.137193	G	G	0.4911		
rs35213536	20	62694319	0.261267	T	T	0.1884		
rs62229372	21	37692507	0.124809	T	T	0.1446		
rs117870289	21	39983448	0.01029	T	T	0.7768		
rs12627651	21	44760603	0.310186	A	A	0.215		
rs9306160	21	45107562	0.380953	T	C	0.1687		
rs4823006	22	29451671	0.428508	G	A	0.1396		
rs9609429	22	32517431	0.269608	C	T	0.1203		
rs139055942	22	40714356	0.07135	G	C	0.3345		

*the reported minor allele frequency (MAF) is computed from MDC; §Beta refers to the one reported in the reference GWAS studies

Table S3 Descriptive characteristics of MPP population

	MPP (n=9,367)	MDC (n=29,295)
	Mean±SD/%	*Mean±SD/%*
Age, yrs	46.8±6.28	58.0±7.61
Sex, % Male	55.4	39.7
BMI, kg/m^2	24.3±3.4	25.8±4.0
Systolic Blood Pressure, mmHg	126.5±14.2	141.1±20.0
Diastolic Blood Pressure, mmHg	85.2±8.7	85.6±10.4
Heart rate, bpm	68.2±9.4	-
Diabetes at baseline, %	3.8	17.4
Smoke, % smokers	32.7	62.1
Hypercholesterolemia, % over 200 mg/dl	66.3	-
HDL cholesterol, mmoL/L	-	4.02±0.43
Hypertensive, %	35.3	61.3
Civil state, % Married/cohabiting	32.7	-
Physical Activity, % Prevalent sedentary habits	32.2	-
GFR, mL/min/1.73m^2	85.3±17.4	-
Apo A, mg/dl	-	156.8±28.2
Apo B, mg/dl	-	107.1±26.1

Legend: APO A: apolipoprotein A; APO B: apolipoprotein B; BMI: body mass index; BP-GRS$_{858}$: Genetic risk score for blood pressure traits made up by 858 SNPs; GFR: estimate glomerular filtration rate; HDL: high density lipoprotein; MDC: Malmö Diet and Cancer; MPP: Malmö Preventive Project; SD: standard deviation

Table S4 BP-GRS$_{858}$ boundaries in different quartiles and deciles in the MDC and MPP cohorts

**n (MDC/MPP)**	**MDC (n=29,295)**	**MPP (n=9,367)**
**Quartiles**		
1st quartile n=7,447/2,305	0.1075-0.1162	0.1079-0.1161
2nd quartile n=7,067/2,373	0.1162-0.1178	0.1161-0.1178
3rd quartile n=7,497/2,372	0.1178-0.1195	0.1178-0.1195
4th quartile n=7,284/2,317	0.1195-0.1279	0.1195-0.1275
**Deciles**		
1st decile n=3,778/948	0.10750.1146	0.1079-0.1146
2nd decile n=3,001/957	0.1146-0.1157	0.1146-0.1157
3rd decile n=1,914/912	0.1157-0.1165	0.1157-0.1165
4th decile n=3,032/980	0.1165-0.1172	0.1165-0.1172
5th decile n=2,771/881	0.1172-0.1178	0.1172-0.1178
6th decile	0.1178-0.1185	0.1178-0.1185

n=3,191/1,009		
7th decile n=2,226/857	0.1185-0.1191	0.1185-0.1191
8th decile n=3,707/955	0.1191-0.1199	0.1191-0.1199
9th decile n=2,702/936	0.1199-0.1210	0.1199-0.1210
10th decile n=2,973/932	0.1210-0.1279	0.1210-0.1275

Legend: MDC: Malmö Diet and Cancer; MPP: Malmö Preventive Project.

Table S5 Comparison of the association between BP-GRS*858* and systolic blood pressure (SBP) and diastolic blood pressure in the MDC

SBP, mmHg				DBP, mmHg		
Model A (r^2=0.24)*				Model A (r^2=0.14)*		
	Beta (95%CI)	*p-value*	*partial r*	*Beta (95%CI)*	*p-value*	*partial r*
**Age, ys**	1.05 (1.02-1.08)	$1.00x10^{-300}$	0.41	0.17 (0.16-0.19)	$7.02x10^{-109}$	0.14
**Sex**	-2.51 (-2.94-(-2.07))	$1.19x10^{-29}$	-0.07	-3.43 (-3.68-(-3.21)	$2.39x10^{-176}$	-0.02
**BMI†**	3.44 (3.22-3.66)	$1.30x10^{-209}$	0.19	0.22 (2.19-2.41)	$1.00x10^{-300}$	0.23
**BP-GRS$_{858}$†**	3.26 (3.05-3.47)	$7.46x10^{-200}$	0.18	1.74 (1.63-1.86)	$5.07x10^{-206}$	0.18
Model B (r^2=0.24)*				Model B (r^2=0.15)*		
	Beta (95%CI)	*p-value*	*partial r*	*Beta (95%CI)*	*p-value*	*partial r*
**Age, ys**	0.17 (0.16-0.19)	$7.02x10^{-109}$	0.14	0.17 (0.16-0.19)	$7.02x10^{-109}$	0.14
**Sex**	-3.44 (-3.68-(-3.21)	$2.39x10^{-176}$	-0.18	-3.44 (-3.68-(-3.21)	$2.39x10^{-176}$	-0.18
**BMI†**	2.32 (2.20-2.43)	$1.00x10^{-300}$	0.23	2.32 (2.20-2.43)	$1.00x10^{-300}$	0.23
**BP-GRS$_{29}$†**	0.24 (0.12-0.37)	$8.44x10^{-5}$	0.02	0.24 (0.12-0.37)	$8.44x10^{-5}$	0.02
**BP-GRS$_{858}$†**	1.65 (1.53-1.77)	$1.91x10^{-154}$	0.16	1.65 (1.53-1.77)	$1.91x10^{-154}$	0.16

* Model A: age, sex, BMI; model B: as model A plus BP-GRS$_{29}$. †expressed for standard deviation unit increment

Legend: BMI: body mass index; BP-GRS$_{29:}$ blood pressure genetic risk score made up by 29 SNPs; BP-GRS$_{858}$: blood pressure genetic risk score made up by 858 SNPS; DBP: diastolic blood pressure; r^2: r-squared SBP; systolic blood pressure.

Table S6 Comparison of the association between BP-GRS_{858} and systolic blood pressure (SBP) and diastolic blood pressure in the MPP

**SBP, mmHg**				**DBP, mmHg**		
Model A (r^2=0.15)*				Model A (r^2=0.15)*		
	Beta (95%CI)	*p-value*	*partial r*	*Beta (95%CI)*	*p-value*	*partial r*
**Age, ys**	0.48 (0.42-0.54)	**$1.25x10^{-88}$**	0.21	0.229 (0.196-0.262)	**$8.80x10^{-42}$**	0.14
**Sex**	3.26 (2.59-3.92)	**$6.38x10^{-16}$**	0.08	3.62 (3.21-4.04)	**$7.23x10^{-64}$**	0.18
**BMI†**	1.24 (1.15-1.33)	**$2.75x10^{-144}$**	0.26	0.74 (0.68-0.80)	**$1.60x10^{-145}$**	0.26
**BP-GRS_{858}†**	2.61 (2.33-2.90)	**$1.14x10^{-68}$**	0.18	1.72 (1.54-1.90)	**$6.76x10^{-77}$**	0.19
Model B (r^2=0.15)*				Model B (r^2=0.15)*		
	Beta (95%CI)	*p-value*	*partial r*	*Beta (95%CI)*	*p-value*	*partial r*
**Age, ys**	0.55 (0.50-0.61)	**$6.28x10^{-89}$**	0.21	0.230 (0.20-0.26)	**$5.25x10^{-42}$**	0.14
**Sex**	2.79 (2.11-3.47)	**$8.94x10^{-16}$**	0.08	3.61 (3.20-4.03)	**$1.47x10^{-63}$**	0.18
**BMI†**	1.20 (1.11-1.29)	**$2.54x10^{-144}$**	0.26	0.74 (0.68-0.79)	**$1.41x10^{-145}$**	0.26
**BP-GRS_{29}†**	0.42 (0.11-0.72)	**0.01**	0.03	0.29 (0.10-0.47)	**0.003**	0.03
**BP-GRS_{858}†**	2.53 (2.23-2.84)	**$5.36x10^{-60}$**	0.17	1.65 (1.46-1.83)	**$6.52x10^{-67}$**	0.18

* Model A: age, sex, BMI; model B: as model A plus BP-GRS_{29}. †expressed for standard deviation unit increment

Legend: BMI: body mass index; BP-$GRS_{29:}$ blood pressure genetic risk score made up by 29 SNPs; BP-GRS_{858}: blood pressure genetic risk score made up by 858 SNPS; DBP: diastolic blood pressure; r^2: r-squared SBP; systolic blood pressure.

Table S7 Association of BP-GRS_{858} with prevalence of hypertension in the MPP and in the MDC

**Prevalence of hypertension, MPP**						
	Model A* n=9,156		Model B* n=8,804		Model C* n=8,625	
	OR (95% CI)	*p-value for trend*	*OR (95% CI)*	*p-value for trend*	*OR (95% CI)*	*p-value for trend*
**BP-GRS_{858}** For 1 SD increase	1.35 (1.29-1.41)	**$1.28x10^{-37}$**	1.36 (1.30-1.43)	**$5.03x10^{-34}$**	1.21 (1.13-1.29)	**$1.64x10^{-8}$**
**BP-GRS_{858}** 4th vs 1st quartile (44.3% vs 26.3% HT)	2.14 (1.88-2.44)	**$1.91x10^{-31}$**	2.21 (1.92-2.54)	**$6.63x10^{-30}$**	1.58 (1.32-1.90)	**$5.20x10^{-7}$**
**BP-GRS_{858}** 10th vs 1st decile (47.4% vs 23.3% HT)	2.95 (2.39-3.64)	**$1.04x10^{-32}$**	2.90 (2.31-3.63)	**$1.48x10^{-29}$**	1.81 (1.35-2.42)	**$6.72x10^{-6}$**
**Prevalence of hypertension, MDC**						
	Model A* n= 29,262		Model B* n= 26,578		Model C* n= 26,562	
	OR (95% CI)	*p-value for trend*	*OR (95% CI)*	*p-value for trend*	*OR (95% CI)*	*p-value for trend*
**BP-GRS_{858}** For 1 SD increase	1.45 (1.41-1.49)	**$1.2x10^{-171}$**	1.47 (1.43-1.52)	**$3.2x10^{-151}$**	1.14 (1.09-1.19)	**$1.0x10^{-7}$**
**BP-GRS_{858}** 4th vs 1st quartile (71.9% vs 50.8% HT)	2.78 (2.56-3.01)	**$6.4x10^{-150}$**	2.88 (2.64-3.14)	**$1.55x10^{-138}$**	1.42 (1.22-1.64)	**$2.47x10^{-5}$**
**BP-GRS_{858}** 10th vs 1st decile (74.7% vs 48.3% HT)	3.72 (3.32-4.14)	**$4.2x10^{-143}$**	3.89 (3.45-4.39)	**$3.27x10^{-145}$**	1.67 (1.36-2.04)	**$2.50x10^{-5}$**

**See methods in the main text for different covariates added in different regression models*

Legend: BP-$GRS_{29:}$ blood pressure genetic risk score made up by 29 SNPs; BP-GRS_{858}: blood pressure genetic risk score made up by 858 SNPS; OR: Odd Ratio

Supplementary Figures

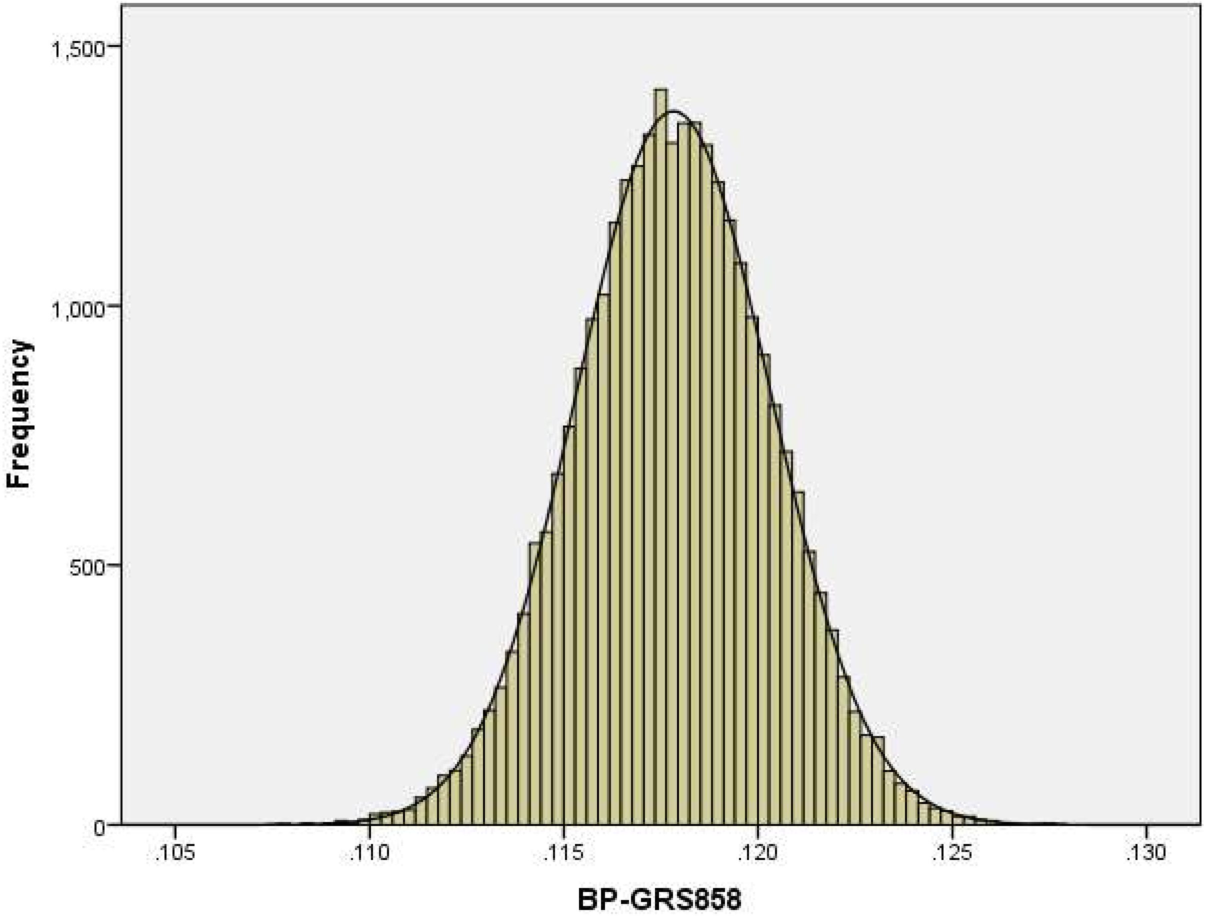

Figure S1. BP-GRS_{858} in MDC

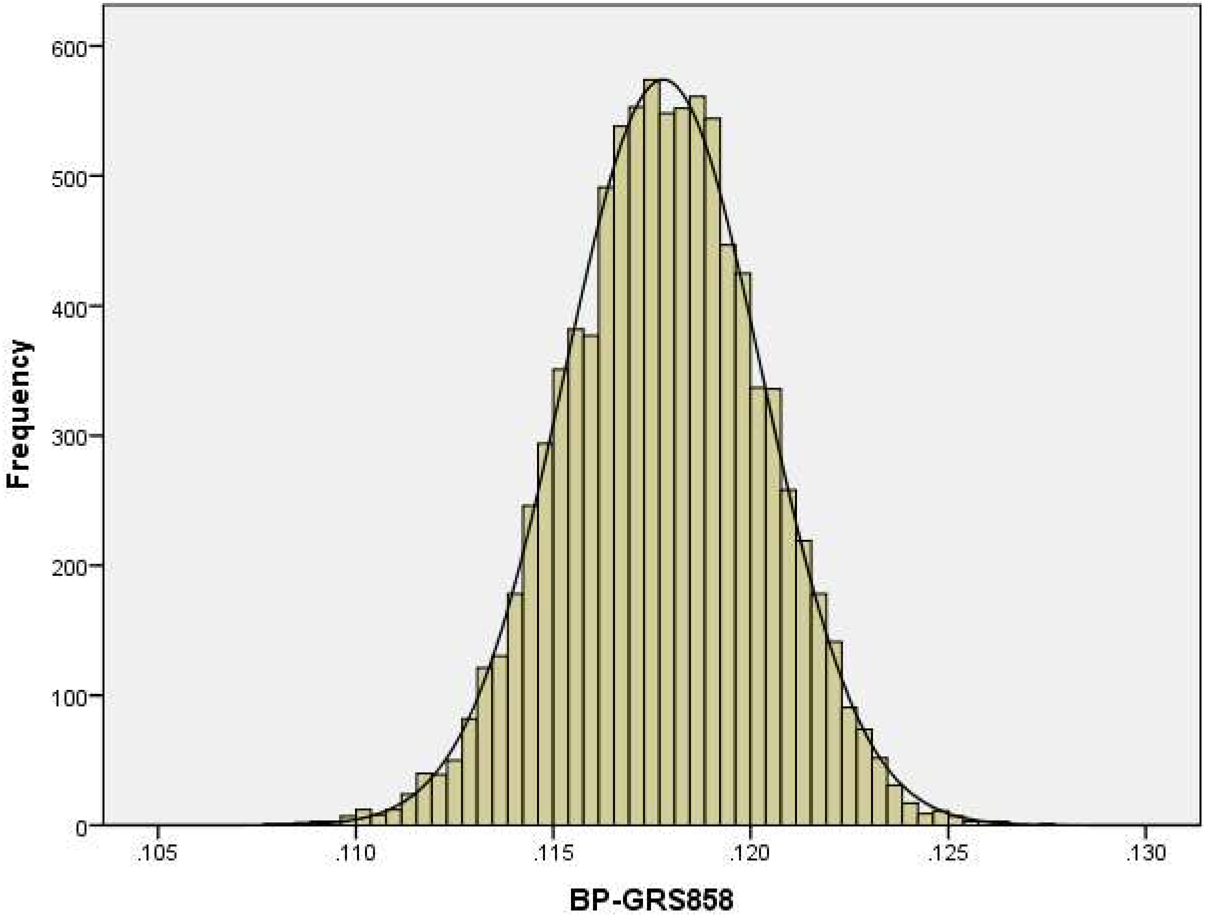

Figure S2. BP-GRS$_{858}$ in MPP

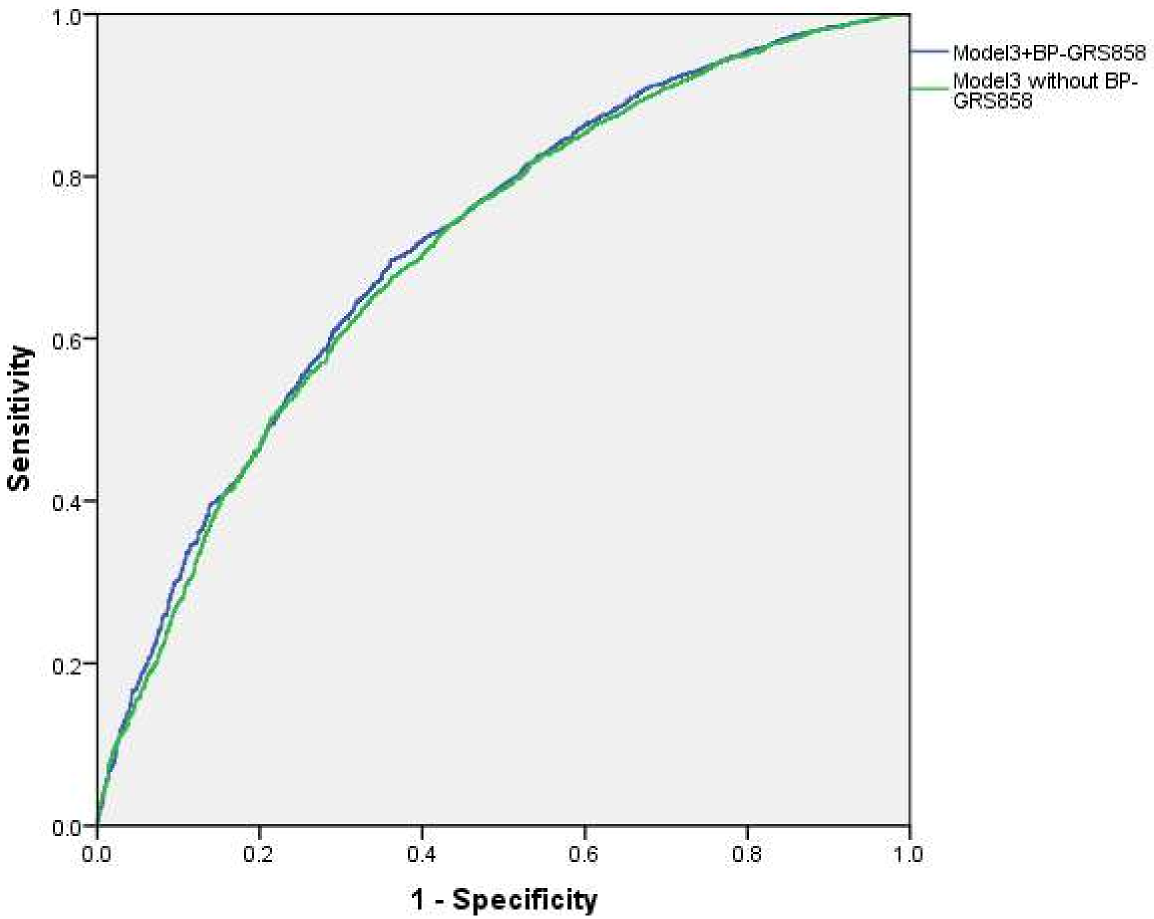

Figure S3. ROC curve of model 3 with (green line) and without (blue line) adding BP-GRS_{858}
